# Multi-Noncentrosymmetric Polar Order in 2D Hybrid Lead Chloride with Broadband Emission and High-Temperature Second-Harmonic Generation Switching

**DOI:** 10.1021/acsami.4c14244

**Published:** 2024-10-24

**Authors:** Mirosław Mączka, Jan K. Zaręba, Anna Gągor, Katarzyna Fedoruk-Piskorska, Dagmara Stefańska, Dawid Drozdowski, Maciej Ptak, Adam Sieradzki

**Affiliations:** † W. Trzebiatowski Institute of Low Temperature and Structural Research of the Polish Academy of Sciences, Okólna 2, 50-422 Wroclaw, Poland; ‡ Institute of Advanced Materials, Faculty of Chemistry, Wrocław University of Science and Technology, Wybrzeże Wyspiańskiego 27, 50-370 Wrocław, Poland; § Department of Experimental Physics, Wrocław University of Science and Technology, Wybrzeże Wyspiańskiego 27, 50-370 Wrocław, Poland

**Keywords:** ferroelectricity, hybrid, lead, perovskite, 3-chloropropylammonium

## Abstract

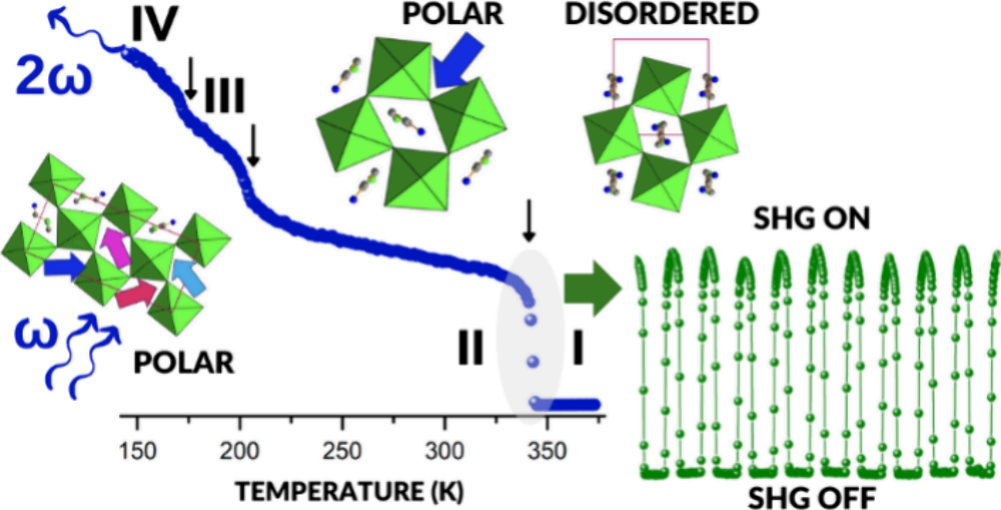

Two-dimensional lead halide perovskites represent a fascinating class of hybrid semiconductors for solar cell, light-emitting, nonlinear optical (NLO), and ferroelectric applications. A notable subset within this category is luminescent ferroelectrics, which have garnered considerable attention for their potential in integrated photoelectronic devices. In this study, we employed an organic amine halogenation strategy (also referred to as halogen engineering), which is renowned for its efficacy in inducing polar order through crystal engineering. Consequently, we synthesized a layered Ruddlesden–Popper (RP) lead chloride comprising 3-chloropropylammonium cations (CPA^+^), with the chemical formula CPA_2_PbCl_4_. This compound features as many as four temperature-dependent crystal phases, with phase transitions observed at *T*_1_ = 353.1 K (343.9 K), *T*_2_ = 211.7 K (208.6 K), and *T*_3_ = 182.0 K (178.2 K) in the heating (cooling) cycles. Employing a multitechnique approach—including thermal analysis, X-ray diffraction, dielectric and pyroelectric current measurements, Raman spectroscopy, and second-harmonic generation (SHG) studies—we determined the mechanisms of the structural phase transitions. Our findings demonstrate polar order of phase **II** (space group *Cmc*2_1_), phase **III** (space group *Pna*2_1_), and phase **IV** (space group *Pca*2_1_), while also confirming the centrosymmetric nature of phase **I** (space group *Cmce*). X-ray diffraction data revealed that the **I** to **II** PT is of a ferroelectric nature, devoid of ferroelastic strain, a conclusion further supported by pyroelectric measurements. CPA_2_PbCl_4_ features negative linear thermal expansion and broadband emission, which transitions to white light above 180 K. Remarkably, CPA_2_PbCl_4_ also demonstrates high-temperature SHG on–off switching with a high contrast ratio of 300:1 along with good switching stability, as evidenced by SHG cycling studies at heating/cooling rates ranging from 5 to 50 K/min. This SHG study also sets new standards for the field of SHG switching by providing a method to quantify the thermal responsiveness of SHG-switchable materials using the *t*_req_ (time requirement) parameter. Overall, our findings show that the halogenation strategy has led to the discovery of a rare example of an RP perovskite exhibiting coexistence of white-light emission, SHG *on–off* thermal bistability, ferroelectricity, and negative linear thermal expansion.

## Introduction

Hybrid organic–inorganic lead halide perovskites have garnered enormous interest in recent years due to their remarkable structural versatility and excellent optoelectronic properties.^1−3^ Among these, two-dimensional (2D) layered analogues of general formulas A_2_PbX_4_ and A’PbX_4_, where A and A’ denote monovalent and divalent organic cations, respectively, and X stands for halogen anions, stand out as a particularly intriguing subclass.^4−6^ These compounds are characterized by their unique architecture, consisting of inorganic perovskite layers interspersed with organic cations, effectively functioning as natural quantum wells with 2D electronic confinement within the perovskite layers. The stark contrast in dielectric permittivity between the organic and inorganic components engenders a pronounced dielectric confinement effect. This feature results in a substantial widening of the band gap and a concomitant increase of exciton binding energy compared to their three-dimensional (3D) counterparts.^4,5,7,8^ While this characteristic renders 2D perovskites less suitable for photovoltaic applications, it opens up manifold opportunities in light-emitting applications.^8−10^ In this respect, photoluminescence (PL) associated with free exciton (FE) recombination is narrow and exhibits high color purity,^8,9^ making them prime candidates for light-emitting diode (LED) fabrication.^11^ Moreover, certain 2D perovskites exhibit broadband emission, including white light, a property highly relevant in lightning and display industries.^10^ This type of strongly Stokes-shifted emission occurs due to strong electron–phonon interactions, enabling the self-trapping of excitons.^9,10^ PL attributed to self-trapped excitons (STEs) is usually observed for corrugated perovskites,^9,10,12,13^ but it has also been reported for many (100)-derived analogues.^5,9,14−16^ It should be noted that the intensity of STE-related PL increases in the order Cl > Br > I.^14^

2D perovskites allow the accommodation of various organic spacers, leading to vast structural diversity and tunability of their physicochemical properties. Compounds crystallizing in noncentrosymmetric structures are particularly appealing, as the absence of an inversion center is a prerequisite for second-order nonlinear optical (NLO) properties (e.g., second-harmonic generation, SHG) and for pyro-, piezo-, and ferroelectricity, properties of paramount importance for practical applications. An additional degree of practical utility is offered by SHG switching functionality, as the capacity to control the transition between SHG states of disparate intensity (on, off, and intermediate) through temperature changes renders these materials highly valuable for advanced optical technologies.^17,18^

The number of discovered 2D lead halide perovskites exhibiting NLO and/or ferroelectric properties continues to grow steadily,^19−21^ yet among (100)-derived chlorides, ferroelectricity or SHG activity has been reported for only six analogues, i.e., BZA_2_PbCl_4_, (2-FBZA)_2_PbCl_4_, (4-ClBZA)_2_PbCl_4_, BA_2_PbCl_4_, MHy_2_PbCl_4_, and (*i*-BA)_2_PbCl_4_ (BZA = benzylammonium, 2-FBZA = 2-fluorobenzylammonium, 4-ClBZA = 4-chlorobenzylammonium, BA = butylammonium, and MHy = methylhydrazinium).^20,22−25^ Notably, none of these materials have been investigated for SHG switching capabilities, leaving a significant gap in harnessing their full functional potential.

From an application standpoint, it is highly desirable if a material exhibits two or more functional properties. Luminescent ferroelectrics, which have garnered attention for their potential in integrated photoelectronic devices, represent one such group of materials. Among A_2_PbCl_4_ chlorides, the coexistence of ferroelectricity and narrow UV PL near 360 nm has been reported for BZA_2_PbCl_4_,^26^ while Ji et al. reported in 2019 that BA_2_PbCl_4_ is the first 2D hybrid perovskite ferroelectric exhibiting broadband white-light emission.^27^

Recent literature highlights that the employment of halogenated ammonium cations is a promising strategy for inducing polar order in hybrid perovskites.^20,24,25^ An interesting example is 3-bromopropylammonium (BPA), which was used for the synthesis of BPA_2_PbBr_4_, a bromide exhibiting ferroelectric properties.^28,29^ Ferroelectric properties have also been reported for the lead-free double perovskite comprising the CPA^+^ cation, i.e., for CPA_4_AgBiBr_8_,^30^ yet hitherto remained unexplored in the context of lead halide perovskites.

As part of our extensive research into polar hybrid perovskites, we employed an amine chlorination strategy to facilitate the formation of polar two-dimensional lead halides. Indeed, the successful self-assembly of the CPA^+^ derivative with lead chloride has led to the identification of a unique instance of an amine-halogenated 2D RP hybrid perovskite, CPA_2_PbCl_4_, displaying as many as three acentric crystal phases. The underlying mechanisms of structural phase transitions, along with lattice dynamics and phonon, have been determined using temperature-dependent Raman and dielectric spectroscopies.

We highlight the functional properties of CPA_2_PbCl_4_ that are related or arise directly from its rich noncentrosymmetry and polarity: multilevel SHG activity due to multiple acentric crystal phases, ferroelectricity accompanied by white-light emission, and high-temperature SHG on–off switching capability. Finally, our SHG temperature cycling study also sets a new benchmark for the field by employing a quantitative method to evaluate the thermal responsiveness of SHG-switchable materials using the *t*_req_ (time requirement) parameter, establishing a new standard for meaningful comparisons of the performance of SHG-switchable materials.

## Results and Discussion

### DSC

**Figure 1:**
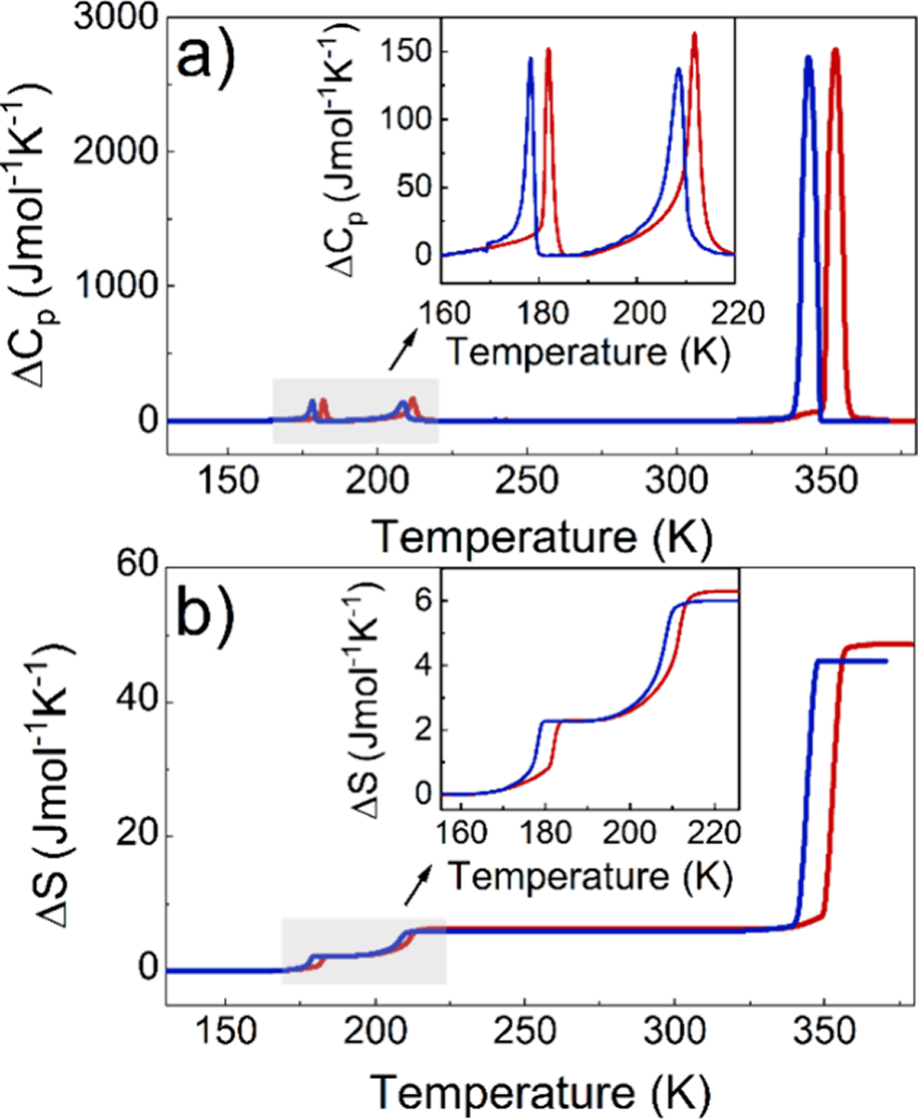
Changes in (a) heat capacity (Δ*C*_p_) and (b) entropy (Δ*S*) related to PTs during the heating (red) and cooling (blue) runs for CPA_2_PbCl_4_.

DSC measurements revealed four reversible PTs at *T*_1_ = 353.1 K (343.9 K), *T*_2_ = 211.7 K (208.6 K), and *T*_3_ = 182.0 K (178.2 K) during heating (cooling) (Figures 1a and S1). The anomaly at temperature *T*_1_ shows strongly symmetric changes of Δ*C*_p_ and discontinuous changes of Δ*S*, combined with thermal hysteresis, confirming the first-order character of this PT. In contrast, the low-temperature (LT) PTs exhibit less intense and symmetrical Δ*C*_p_ changes with smaller temperature hysteresis, suggesting a less pronounced first-order character of these PTs. The associated changes in entropy (enthalpy) are estimated to be ∼42.2 J mol^–1^K^–1^ (14.8 kJ mol^–1^) (*T*_1_), ∼4.0 J mol^–1^K^–1^ (0.77 kJ mol^–1^) (*T*_2_), and ∼2.3 J mol^–1^K^–1^ (0.4 kJ mol^–1^) (*T*_3_) (Figure 1b). The large value of Δ*S* at *T*_1_ points to an order–disorder character of the HT PT, whereas the order of magnitude smaller values of Δ*S* at *T*_2_ and *T*_3_ suggest that all the LT PTs have a displacive character.

### Single-Crystal X-ray Diffraction

CPA_2_PbCl_4_ belongs to the large family of RP 2D perovskites with the formula A_2_BX_4_, where A is a monovalent cation, and X stands for a halide.^31,32^ The single [PbCl_4_]_*n*_^2–^ layers represent (100)-oriented perovskite slabs. These slabs alternate with protonated organic cations, balancing the effective charges. In the case of protonated amines, aside from the Coulomb interactions, the organic part interacts with the inorganic layers through the N–H···X hydrogen bonds. For halogenated molecules, additional forces stabilizing the crystal structure are halogen–halogen interactions. These noncovalent forces enhance the stability and performance of perovskite solar cells by increasing hydrophobicity and reducing surface trap states.^33−35^

In CPA_2_PbCl_4_, all the mentioned interactions influence the crystal structure and are activated with temperature, leading to rich, temperature-induced polymorphism. The material possesses four polymorphic phases and undergoes group-subgroup-related PTs from the centrosymmetric HT orthorhombic *Cmce* symmetry (phase **I**) to polar phases of *Cmc*2_1_ (phase **II**), *Pna*2_1_ (phase **III**), and *Pca*2_1_ (phase **IV**) space groups (see crystal data in Tables S1–S3). The first PT is of the *translationengleiche* (*t*-type), breaking symmetry (i.e., involving the loss of the center of symmetry in phase **II**) so that a permanent dielectric dipole moment along the *c* direction is induced. This PT does not alter the translation symmetry, and the unit cell dimensions are preserved in low-symmetry phase **II**. The PT from phases **II** to **III** is of the *k*-type (*klassengleichhe*) and involves changes in translation symmetry along the *b* direction, which is doubled. Thus, phases **III** and **IV** accommodate a unit cell with a volume twice that of phases **I** and **II**, which is also related to the loss of C-centering in the unit cell. The last, third phase transition is of the *t*-type.

Phase **II** is a maximal nonisomorphic subgroup of *Cmce*, whereas the remaining LT phases (**III** and **IV**) are both maximal nonisomorphic subgroups of phase **II** (*Cmc*2_1_). Additionally, the **I** to **II** PT is related to the *mmmFmm*2 symmetry reduction and is of ferroelectric nature, without the formation of ferroelastic strain.^36^

The HT phase **I** of CPA_2_PbCl_4_ exhibits significant disorder in both the organic and inorganic components, resulting in split atoms of CPA^+^ and large displacement parameters for all atoms. Despite employing a two-state model for the amine, which is disordered at two equivalent positions with 0.5 occupancy to represent the diffuse electron density between the inorganic slabs, the large displacement parameters and very large PT entropy suggest a potential for free rotations along molecular axes and conformational changes.

The transition to the *Cmc*2_1_ phase is predominantly of an order–disorder nature, accompanied by slight deformation of the inorganic layers. This phase is characterized by an increase in in-plane octahedral distortion from 28 to 36°, while the out-of-plane deviation remains equal at 0° in phases **I** and **II**. The driving force behind this PT is the emergence of Cl···Cl halogen interactions in a parallel displaced geometry between the halogenated CPA^+^ and Cl^–^ ligands, with a C–Cl···Cl distance of 3.62 Å and a C–Cl–Cl angle of 172° at 295 K. The significant reduction in C–Cl···Cl distances (from 5.07 Å in **I**) affects the interlayer voids, leading to a marked decrease in the lattice parameter perpendicular to the layers, from 28.552(7) Å at 360 K to 26.725(7) Å at 295 K. These changes are accompanied by an increase in in-plane distances, resulting in linear negative thermal expansion in the *b* direction. The lattice parameter *b* increases from 7.855(3) Å at 360 K to 8.019(3) Å at 295 K, with further extension observed as the temperature decreases. Figure S2 illustrates the temperature evolution of lattice parameters in the subsequent phases.

**Figure 2:**
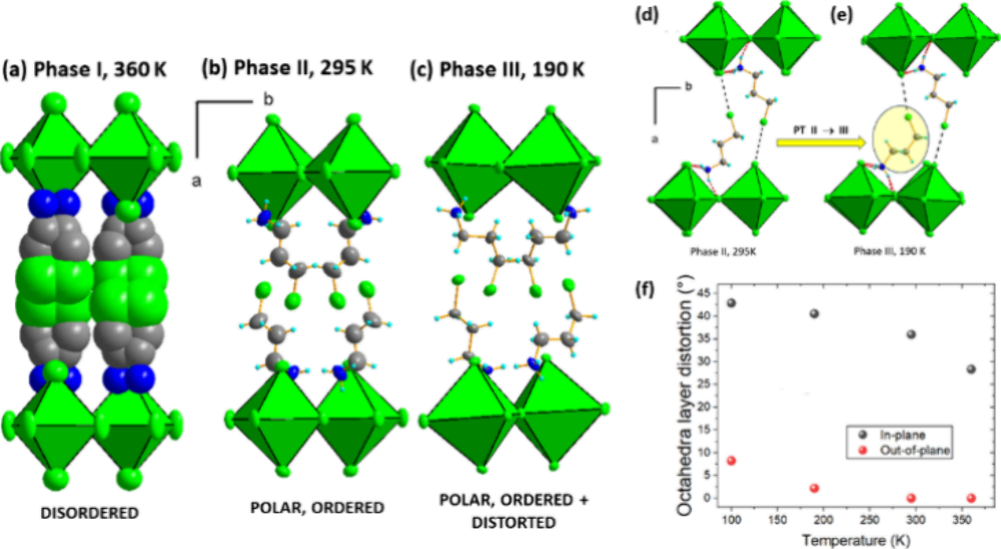
(a–c) Structural changes in inorganic slabs and interlayer organic in CPA_2_PbCl_4_ for subsequent phases. (d, e) Conformational changes of CPA^+^ during the PT from phase **II** to phase **III**. (f) Distortions among the layers of the octahedra. Displacement parameters are shown at the 50% probability level.

Figure 2 depicts the structural changes in the inorganic slabs and interlayer organic cations in CPA_2_PbCl_4_ for temperature-induced crystal phases. In phase **II**, the molecular structure is ordered with a single CPA^+^ adopting an *anti*-conformation which is anchored between neighboring layers through N–H···Cl and C–Cl···Cl. Further cooling strengthens noncovalent interactions within the structure and induces conformational changes among the CPA^+^ units. In phase **III**, four independent ammonium cations are present, with two adopting the *anti*-conformation and the remaining transitioning to *gauche* conformers through rotation of the NH_3_–C2 terminal group along the internal C–C bond (see Figure 2d,e). The increase in the strength of N–H···Cl hydrogen bonds is evidenced by out-of-plane distortions of the octahedra and further growth of in-plane deformations (Figure 2f). In phase **III**, the inorganic component consists of corner-sharing Pb(1)Cl_6_ and Pb(2)Cl_6_ octahedra, with the asymmetric unit comprising two lead atoms, four apical chlorine atoms, and four bridging chlorine atoms.

The final PT, from phase **III** to **IV,** involves additional rearrangements of the molecular part and structural distortions of [PbCl_4_]_*n*_^2–^. The change of symmetry from *Pna*2_1_ to *Pca*2_1_ entails a new distribution of symmetry-independent CPA^+^ cations within the structure (details are shown in Figure S3) and a decrease in the symmetry of the layers. Phase **IV** features two independent [Pb(1)Cl_4_]_*n*_^2–^ and [Pb(2)Cl_4_]_*n*_^2–^ layers, composed exclusively of Pb(1)Cl_6_ and Pb(2)Cl_6_ octahedra. In phase **IV**, the C–Cl···Cl bond lengths range between 3.31 and 3.77 Å, whereas the donor-to-acceptor distances in hydrogen bonds range between 3.151(6) and 3.354(6) Å. The increase in the strength of hydrogen and halogen interactions forces the octahedra to deform. The bond length distortion of PbCl_6_ grows from 0.2 (**I**) < 1.7 (**II**) < 4.4 (**III**) < 5.6 (**IV**), and the angle variance (σ^2^ [deg.^2^]) increases from 0 (**I**) < 28 (**II**) < 39 (**III**) < 50 (**IV**). Phase **IV** is also characterized by the largest in-plane and out-of-plane deviations between the octahedra.

**Figure 3:**
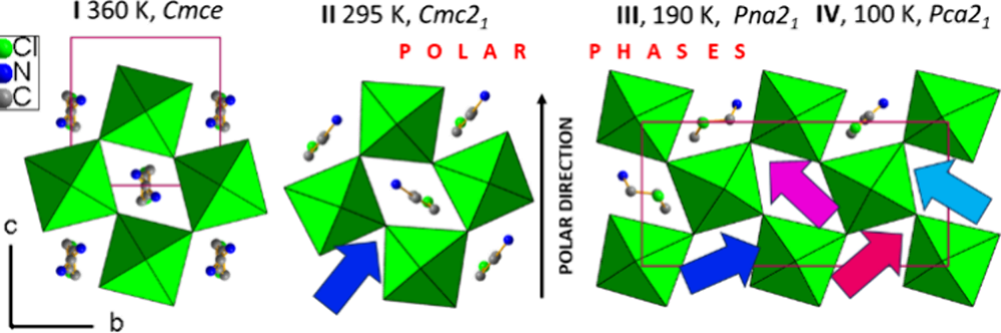
Single [PbCl_4_]_*n*_^2–^ layers with embedded CPA^+^ in subsequent phases. The number of symmetry-independent molecules increases from 0.5 in **I**, to 1 in **II** and 4 in **III** and **IV**. The independent dipole moments of CPA^+^ are depicted as colored arrows. In **III** and **IV**, the unit cell is doubled in the *b* direction.

The LT phases of CPA_2_PbCl_4_ develop in-plane spontaneous polarization along the *c* direction, as depicted schematically in Figures 3 and S4. The primary component of this polarization arises from the internal dipoles of molecular CPA^+^ counterions.

### Temperature-Dependent Raman Study

**Figure 4:**
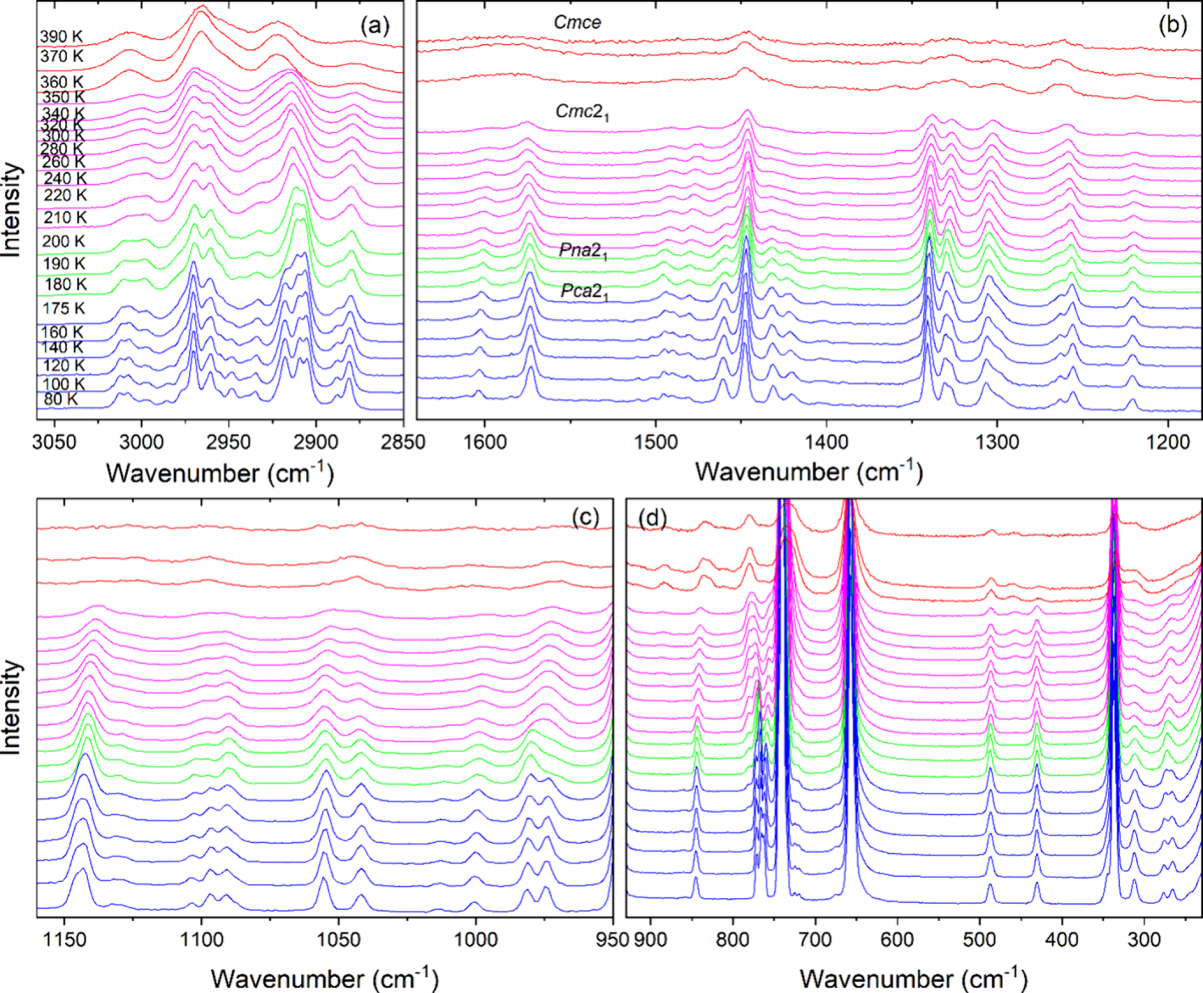
Raman spectra of CPA_2_PbCl_4_ in the (a) 3050–2850 cm^–1^, (b) 1640–1180 cm^–1^, (c) 1160–950 cm^–1^, and (d) 930–230 cm^–1^ ranges.

To monitor the effect of temperature on lattice dynamics and obtain further insights into mechanism of the PTs, Raman spectroscopy was performed in the 80–390 K range (Figures 4, S5 and S6). The observed vibrational modes are listed in Table S4 together with the assignments based on vibrational studies and theoretical calculations of *n*-propylamine and 1-chloropropane.^37,38^

At temperatures exceeding 350 K, the Raman spectra exhibit broad bands, especially those related to the N–H stretching and bending modes (Figures 4 and S5). This behavior proves that the organic cations are disordered in phase **I**.

The PT at *T*_1_ is marked by abrupt narrowing and shifts of the Raman bands corresponding to the internal vibrations of CPA^+^. The narrowing is especially pronounced for bands related to NH_3_ group vibrations (Figures 4 and S5). For instance, the full width at half-maximum (fwhm) of the ν_as_(NH_3_) and δ_as_(NH_3_) modes decreases from 75.8 and 43.7 + 19.6 cm^–1^ at 360 K to 52.7 and 18.2 + 13.6 cm^–1^ at 350 K. This behavior proves that the PT at *T*_1_ is associated with the ordering of the CPA^+^ cations. Regarding shifts, the δ_as_(NH_3_) modes show negligible shifts from 1598 + 1577 cm^–1^ at 360 K to 1597 + 1576 cm^–1^ at 350 K, but the ν_as_(NH_3_) mode exhibits a significant upshift from 3190 cm^–1^ at 360 K to 3206 cm^–1^ at 350 K. Significant shifts are also observed for many other modes (see, for instance, the ν_s_(NH_3_), ν_s_(CH_2_), and ν_s_(CH_2_) modes, which shift from 3007, 2967, and 2922 cm^–1^ at 360 K to 3000, 2970, and 2917 cm^–1^ at 350 K). This behavior indicates that the ordering of CPA^+^ cations is associated with an increase of the amine-framework interactions via N–H···Cl hydrogen bonds, as evidenced from structural data. In the lattice mode region, the PT at *T*_1_ is evidenced by weak shifts and changes in the relative intensities of Raman bands (Figure S6). For instance, the bands at 179, 55, and 35 cm^–1^ at 360 K shift to 175, 59, and 41 cm^–1^ at 350 K. This behavior is consistent with a small deformation of the inorganic layers during the PT from **I** to **II.**

As the temperature decreases below 350 K, Raman bands narrow due to a decrease of phonon–phonon anharmonic interactions. The PT at *T*_2_ does not lead to any clear change in the fwhm of the Raman bands (Figures 4, S5 and S6), confirming that **II** to **III** PT is not related to any order–disorder phenomena. It also does not lead to any significant shifts in Raman bands, either in the internal and lattice modes regions, although some band intensities change (see, for instance, Raman bands in the 3020–2900 cm^–1^ range, Figure 4a). This behavior suggests that the PT at *T*_2_ has a minimal impact on hydrogen bond strength, amine-framework interactions, as well as the tilts and/or distortion of PbCl_6_ octahedra. Notably, X-ray diffraction data indicate that phase **II** contains only one independent CPA^+^ cation and one PbCl_6_ octahedron, while phase **III** contains four (two) independent CPA^+^ cations (PbCl_6_) octahedra. Therefore, one would expect to observe splitting of internal (lattice) modes into four (two) components on going from phase **II** to phase **III**. An inspection of Table S4 shows that splitting is observed only for a few modes and only into doublets. The lack of more pronounced splitting can be most likely attributed to similar structures of these independent structural entities, resulting in similar Raman wavenumbers that cannot be clearly resolved in phase **III** due to band broadening.

The last PT at *T*_3_ is distinctly observed in the Raman spectra. First, many Raman bands, especially lattice modes and internal modes related to vibrations of the NH_3_ groups, split into several components that become very narrow at 80 K (see Figure S6 for the lattice modes and Figures 4a–c, S5, and Table S4 for the ν_as_(NH_3_), ν_s_(NH_3_), δ_s_(NH_3_), and ρ(NH_3_^+^) modes). Second, many bands exhibit a pronounced increase in intensity (see, for instance, the 2918, 917, 773, or 266 cm^–1^ modes, Figure 4a,c,d). This behavior is consistent with a significant rearrangement of the CPA^+^ cations and a decrease in the symmetry of the inorganic layers, as revealed by X-ray diffraction.

### Dielectric Properties

**Figure 5:**
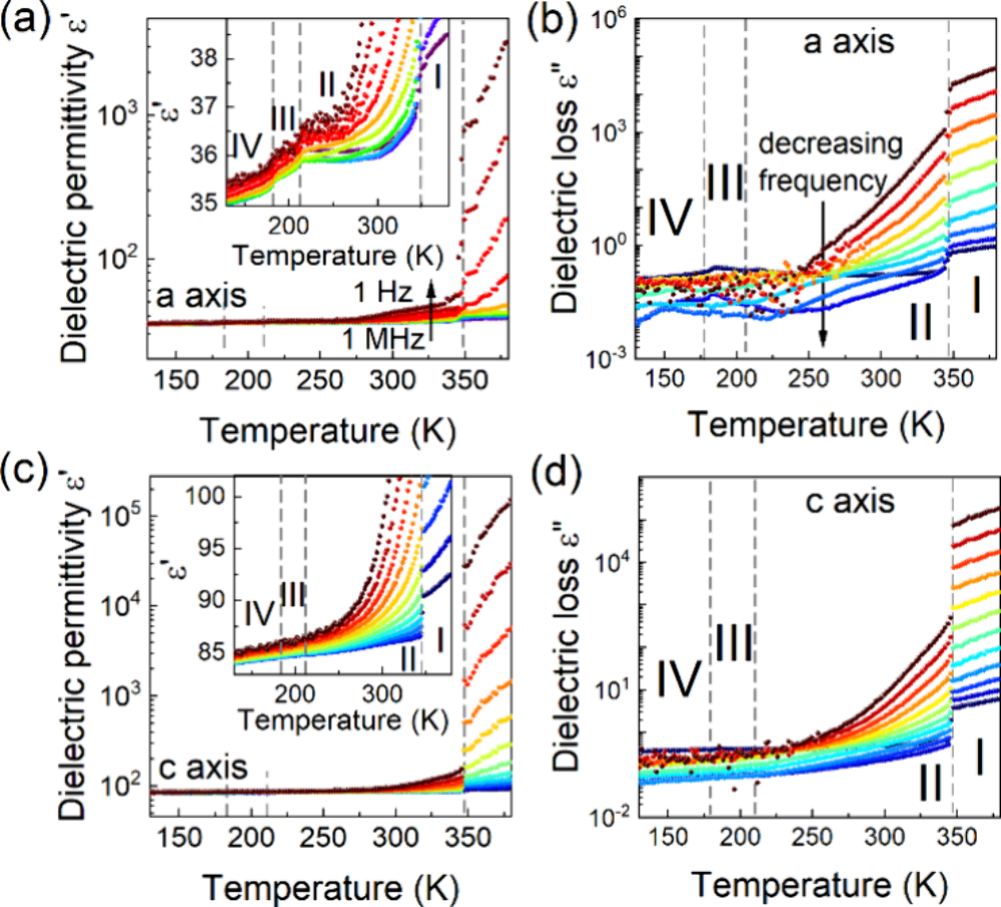
Temperature dependence of real (a and c) and imaginary (b and d) parts of dielectric permittivity measured in single crystals along the *a* and *c* axes.

**Figure 6:**
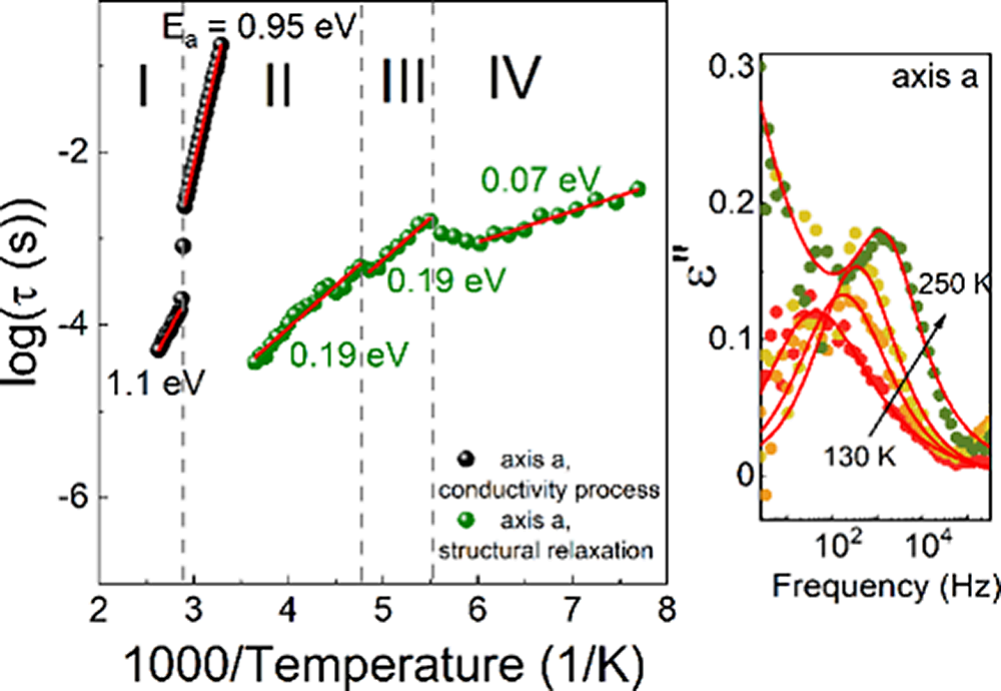
Relaxation map, i.e., log(τ_max_) as a function of 1000/*T*. The right side of the graph shows examples of fits using the Havriliak–Negami function.

Broadband dielectric spectroscopy was used to investigate the changes in the structural dynamics in the vicinity of phase transitions in CPA_2_PbCl_4_. The temperature-dependent real (ε’) and imaginary (ε″) components of the complex dielectric permittivity ε* = ε’ – *i*ε″, measured along two crystallographic directions, are presented in Figure 5. The most significant dielectric anomaly is observed at the phase transition from phase **II** to **I**, measured in the direction of the *a* and *c* axes (Figure 5). This step-like anomaly confirms that the complex dielectric permittivity experiences a kink at *T*_1_, indicating sudden changes in molecular dynamics associated with structural ordering changes. The remaining anomalies at the PTs observed only along the *a* axis are much weaker, which is consistent with the structural changes (Figure 5a). Additionally, a close inspection of the complex dielectric permittivity data in the frequency domain confirms that well-defined dipolar relaxation processes appear in the temperature range from 130 to 274 K for measurements along the *a* axis (Figure S7). At high temperatures (above 275 K), frequency dispersion can be observed, indicating an increase in ionic conductivity. In addition to complex conductivity, the electric modulus representation is also frequently applied for the analysis of the data obtained from conducting systems (M* = 1/ε*). The analysis of modulus curves measured along the *a* and *c* axes demonstrates characteristic behavior, where the real parts of the modulus (*M*’) exhibit an S-shape, and the imaginary parts (*M*″) are bell-shaped (Figure S8). This confirms the occurrence of ionic conductivity processes at HT. The analysis of ε″ and *M*″ curves allowed the fitting of curves related to dielectric relaxations and conductivity processes with a single Havriliak–Negami function. To understand the relaxation and conductivity dynamics, the temperature-dependent behaviors of the dielectric relaxation times as a function of 1000/*T* were compared (Figure 6). These relationships are linear; therefore, the activation energies were estimated using the Arrhenius relationship. The estimated activation energies associated with the dielectric relaxation process measured along the *a* axis are approximately 0.07, 0.19, and 0.19 eV in phases **IV**, **III**, and **II**, respectively (Figure 6). Considering the activation energies of the conduction process, they amount to 0.95 eV (phase **II**) and 1.1 eV (phase **I**) for CPA_2_PbCl_4_ (Figure 6).

**Figure 7:**
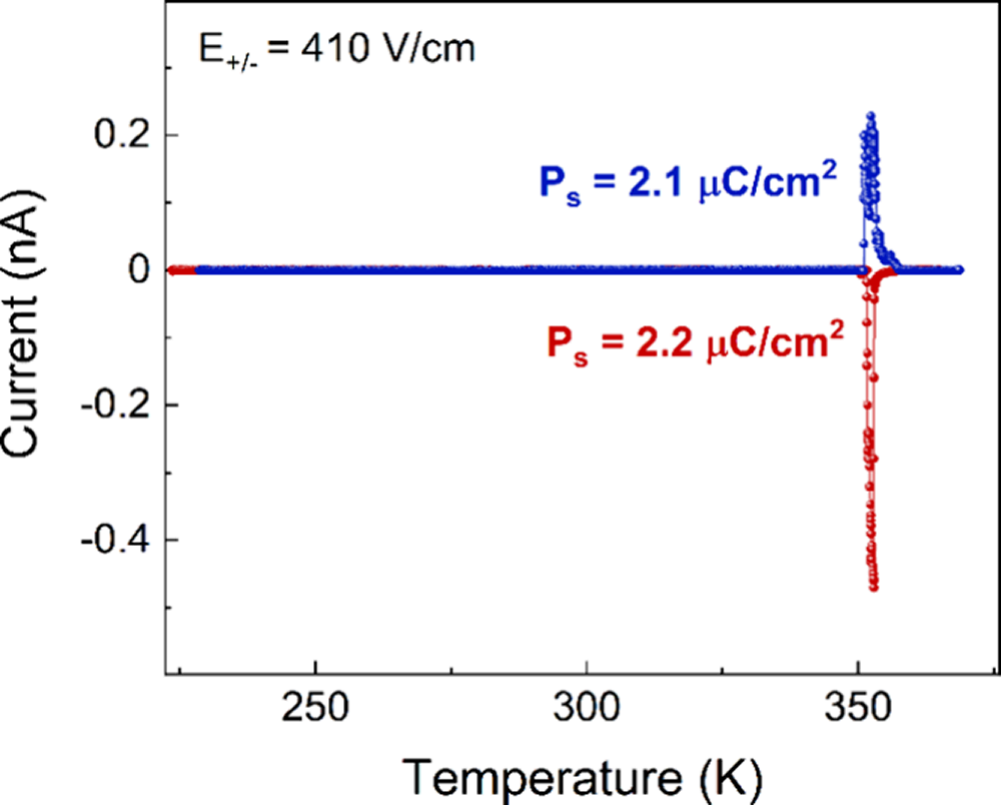
Changes of the pyroelectric current of a single crystal in the direction of the polar axis after poling in the DC electric field.

The temperature-dependent pyroelectric current of a CPA_2_PbCl_4_ single crystal measured along the polar axis is presented in Figure 7. The presence of the pyroelectric current at about 350 K suggests polar behavior in CPA_2_PbCl_4_. Notably, the sign of the current peak changes when reversing the poling field, indicating the ferroelectric nature of the phase transition. By integration of the pyroelectric current, the saturated values of electric polarization were estimated to be approximately 2.2 and 2.1 μC/cm^2^ for positive and negative polarized fields, respectively. To determine whether the observed ferroelectric behavior is of the proper or improper type, we attempted to measure the electric-field dependence of electric polarization in phase **II**. The presence of conductivity prevented the observation of a saturated P–E loop, hindering our ability to ascertain whether the ferroelectric properties of CPA_2_PbCl_4_ are of proper or improper type.

### Linear Optical Properties

The UV absorption spectra of CPA_2_PbCl_4_ span from 200 to 365 nm, featuring two types of bands. The broadband from 200 to 325 nm is directly related to the absorption of individual matrix elements, while a partially overlapping band centered at 338 nm (3.67 eV) corresponds to excitonic absorption (Figure S9). The energy band gap (*E*_g_), determined using a Tauc plot of the absorption edge, equals 3.79 eV (Figure S10). This value is comparable to the *E*_g_ of other (100)-derived lead chlorides comprising small organic cations like methylhydrazinium (MHy_2_PbCl_4_, *E*_g_ = 3.75 eV) or 1,2,4-triazolium (Tz_2_PbCl_4_, *E*_g_ = 3.56 eV).^16,22^

**Figure 8:**
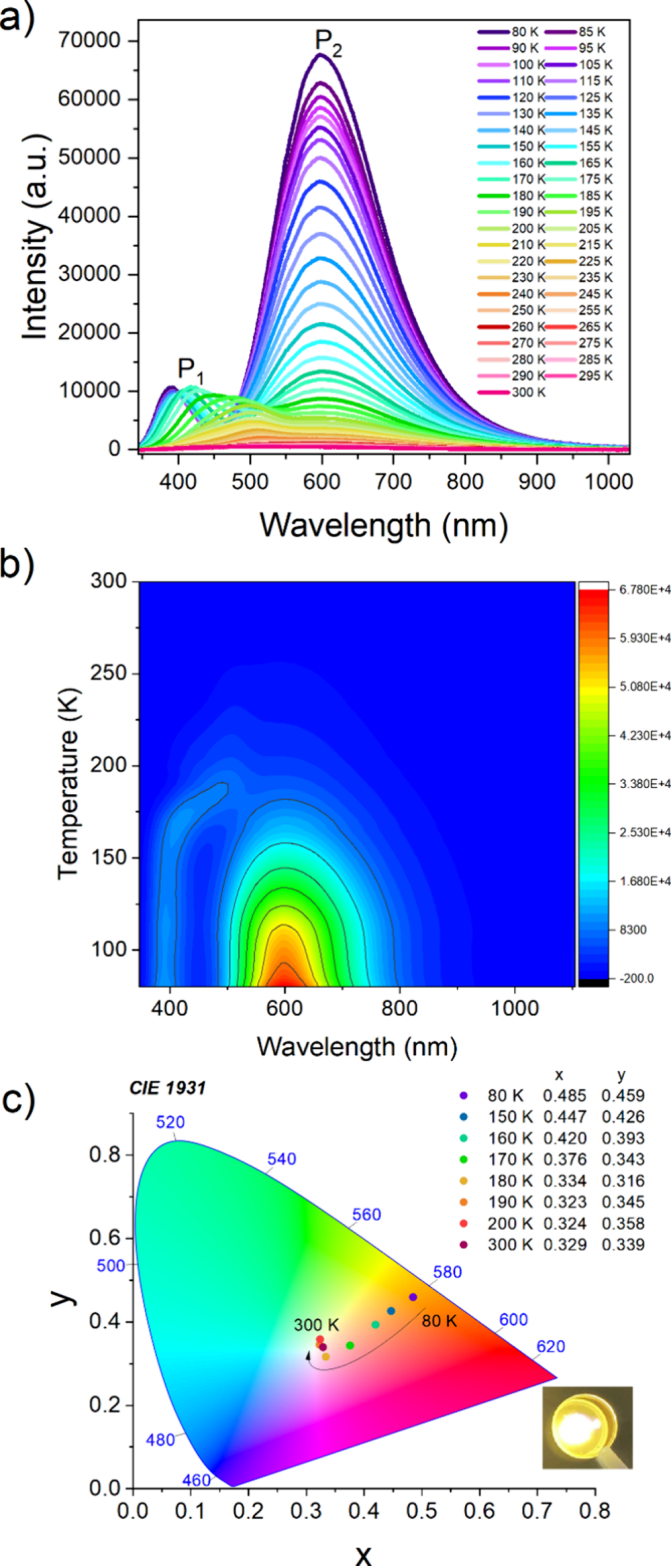
(a) Temperature-dependent emission spectra of CPA_2_PbCl_4_ recorded under 266 nm excitation, (b) temperature dependence of band intensity (contour map), and (c) CIE coordinates of CPA_2_PbCl_4_ at representative temperatures; in the inset the photos of the sample’s emission.

CPA_2_PbCl_4_ exhibits a broadband emission (fwhm = 168 nm) with a maximum at 598 nm and a Stokes shift of approximately 280 nm (P_2_ band in Figure 8a). This broadband PL is typically attributed to radiative recombination from self-trapped excitons (STEs) formed due to local lattice distortion around the excitation site, a phenomenon reported for many 2D perovskites.^14,15,19,39−41^ We tentatively attribute the observed P_2_ band to STEs associated with deep trap states. The emission intensity of P_2_ decreases on heating, with an activation energy (*E*_a_) of 224 meV (Figures S11 and S12), but the band’s position practically does not change over the entire temperature range (Figures 8a,b and S13).

Additionally, CPA_2_PbCl_4_ also shows an additional lower-intensity band centered at 390 nm at 80 K (P_1_ in Figure 8a). The position of the P_1_ band is significantly red-shifted compared to the excitonic absorption band (338 nm) (Figure S9), i.e., its Stokes shift is 52 nm. Furthermore, this band is relatively broad, with an fwhm of 60 nm. The large Stokes shift and fwhm indicate that this PL cannot be attributed to FE but to either STEs localized on shallower trap states (compared to the P_2_ band) or permanent defects. Similar two bands related to STEs were recently observed for 1D aminoguanidinium lead iodide (AGAPbI_3_).^42^ Two bands were also reported for MHy_2_PbCl_4_, but in this case, the higher energy band at 387 nm (at 80 K) was more intense than the lower energy and broader STE-related band at 609 nm.^22^ The location of the P1 band shifts significantly toward higher wavelengths from 406 nm at 160 K to 502 nm at 200 K (Figures 8a,b and S13). This behavior is different from that observed for AGAPbI_3_, for which the higher energy STE band exhibited only a slight shift on heating,^42^ but it resembles the behavior of the higher energy band of MHy_2_PbCl_4_, which shifted from 387 at 80 K to 507 nm at 300 K.^22^ The pronounced shift of the P_1_ band of CPA_2_PbCl_4_ on heating suggests permanent defects as a plausible source of this PL. Another plausible explanation is that this PL originates from STEs, but increasing temperature leads to a gradual population of deeper trap states at the expense of shallower trap states. The integrated emission intensity of the P_1_ band shows nonmonotonous behavior with increasing temperatures in the 155–185 K range (Figure S14). Above 180 K, the PL is quenched with a *E*_a_ of 113 meV (Figure S15).

Due to the strong PL shift with temperature, the color of the observed emission changes from orange-yellow between 80 and 165 K to yellowish-pink at 170 K, and finally to white in the 180–300 K range (Figure 8c).

### Nonlinear Optical Properties and High-Temperature Second-Harmonic Generation Switching Studies

**Figure 9:**
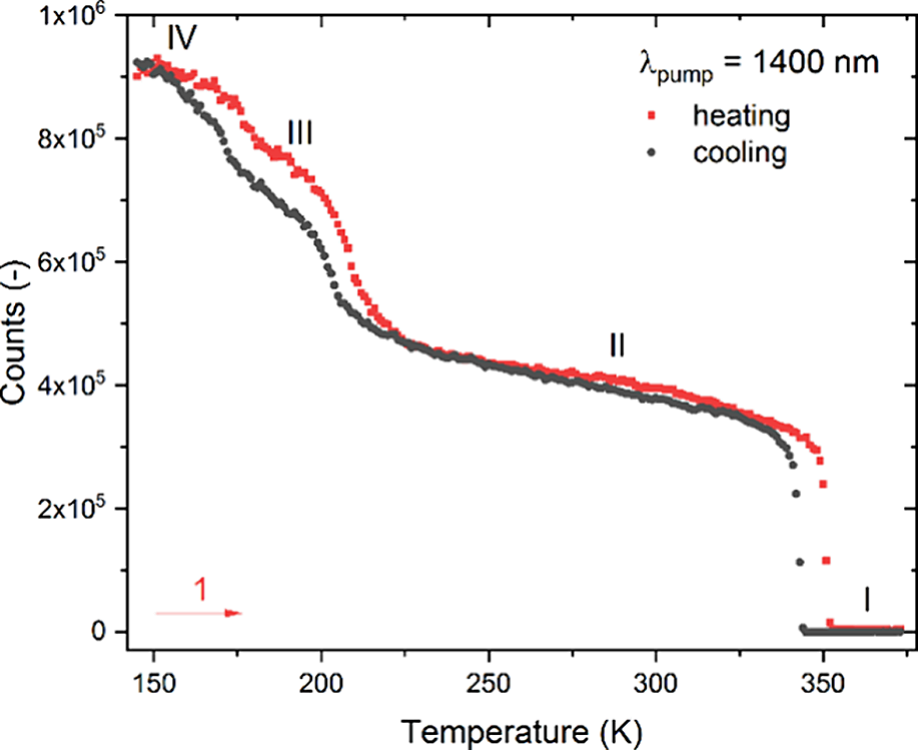
Temperature plot of the SHG integral intensities for CPA_2_PbCl_4_.

A complex PT behavior of CPA_2_PbCl_4_ also calls for SHG screening over a wide temperature range. To this end, SHG studies with 1400 nm femtosecond laser pulses were performed from 178 to 373 K. Figure 9 illustrates the temperature-dependent SHG integral intensities (λ_SHG_ = 700 nm) for both the heating and cooling cycles, with experimental traces of SHG emissions provided in Figure S16. In general, the registered SHG profile shows that three crystal phases (**II**, **III**, and **IV**) exhibit SHG activity, while the HT phase **I** does not, supporting our notion that only this phase among all four identified phases is structurally centrosymmetric. Indeed, upon heating (cooling), one observes at 358 K (348 K) a step-like change in SHG intensity, characterized by a 10 K-wide thermal hysteresis indicative of the first-order nature of the centric-acentric **I** → **II** PT. PTs **II** → **III** and **III** → **IV** are all reflected in SHG profiles, yet they are weakly marked. The calculation of the ratio of integral SHG intensities on either side of PTs **II** → **III** and **III** → **IV** shows that the difference in the SHG intensity between neighboring phases is ca. 20%. This suggests that structural changes responsible for the LT PTs have a rather limited impact on the second-order NLO properties of CPA_2_PbCl_4_. One also sees that these PTs take place at slightly different temperatures during heating and cooling runs, suggesting a first-order character of these PTs.

To gain a semiquantitative picture of the SHG strength of CPA_2_PbCl_4_, a Kurtz–Perry powder test was performed at room temperature (293 K). The relative SHG efficiency of CPA_2_PbCl_4_ is 0.34 that of KDP powder of the same particle size (see Figure S17).

Metal–organic and organic materials displaying step-like changes in SHG intensity due to PT, especially those operating near room or elevated temperatures, have recently gained much attention in the context of SHG switching.^43−48^ Given that CPA_2_PbCl_4_ holds all prerequisites, i.e., features good SHG, and the high-temperature PT occurs from a centrosymmetric to a noncentrosymmetric structure and exhibits first-order characteristics, we explored its SHG switching capabilities. However, a broader comment on the scope and context for this study is necessary. In general, at present, the SHG switching studies present in the literature characterize a few parameters that describe SHG switches’ properties and their eventual relevance for real-life applications. The most commonly characterized are parameters such as the operating temperature, type, and number of distinct SHG states and, in some cases, the SHG contrast. Interestingly enough, the switching properties related to the dynamics of the temperature change are largely neglected. Indeed, little to no information is available about the speed of heating/cooling cycles or how quickly an SHG switch responds to temperature changes. While laboratory setups can precisely control heating/cooling rates, determining the switching speed requires experimental measurement of kinetic parameters that can quantitatively describe the material’s phase-change capability. Quite recently, we arrived at this issue by using a pyrrolidinium-based cyanide perovskite of formula Pirr_2_KCr(CN)_6_ (where Pirr stands for pyrrolidinium), which serves as a case study for bimodal third-harmonic generation and as a dielectric switch.^49^ We there introduced a *t*_req_ (time requirement) parameter, i.e., the time needed for a material to fully transition from one crystal phase to another due to the switching stimulus such as temperature change. *T*_req_ is defined as the ratio of temperature hysteresis (Δ*T*) and the rate of the temperature change (d*T*/d*t*). By employing this parameter, we found that in this material, THG and dielectric switching take place on the single-minute scale, rising exponentially from 1.1 min at a rate of 20 K/min to 3.7 min at 1 K/min. This was by far the first case of quantitative description of the NLO switching speed.

**Figure 10:**
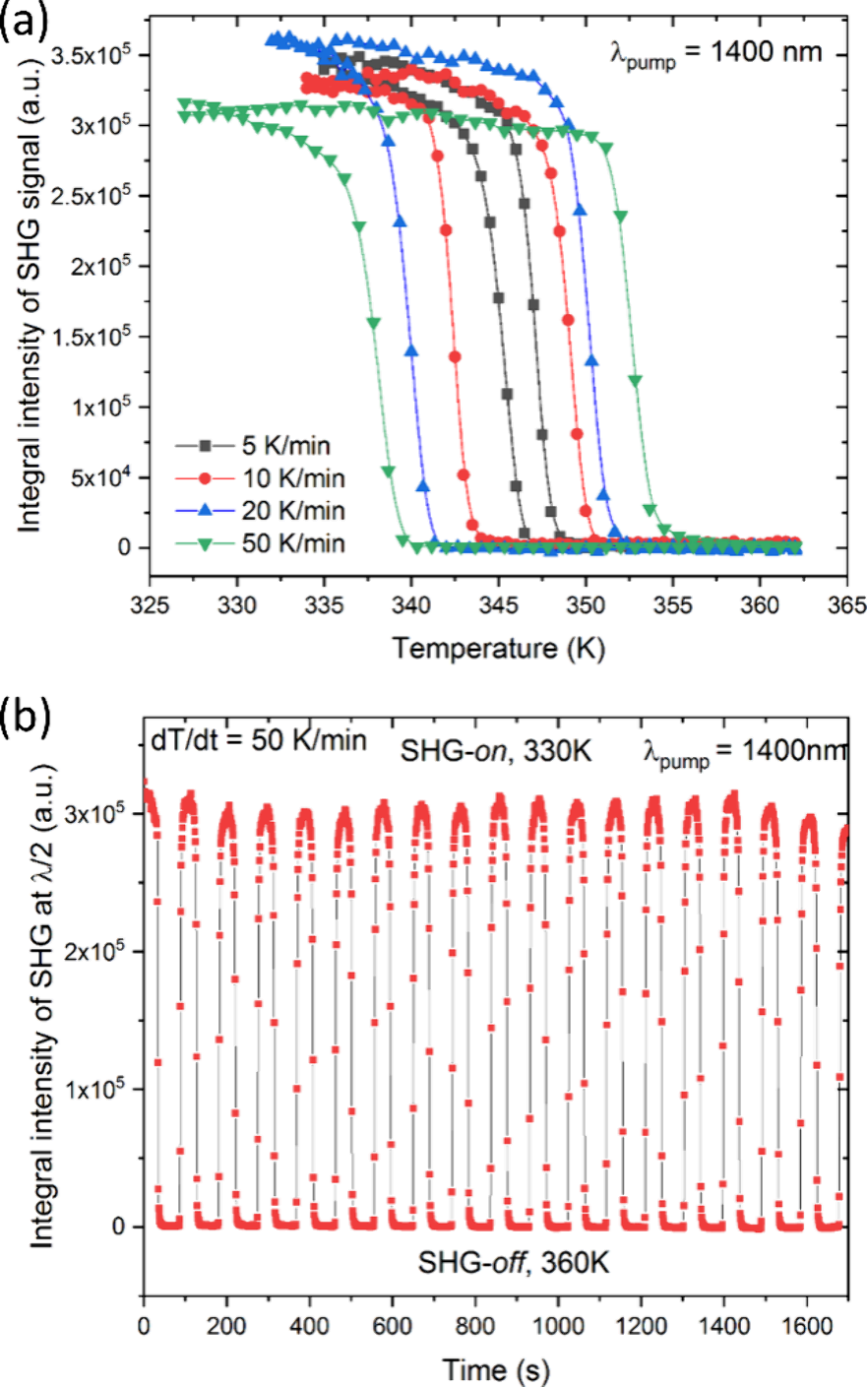
(a) Temperature plot of SHG integral intensities for CPA_2_PbCl_4_ for the **I** → **II** phase transition. (b) Plot of SHG intensity obtained for switching experiment with d*T*/d*t* = 50 K min^–1^ between 330 and 360 K. It should be noted that cycling plots reveal a weak up and down fluctuation of SHG intensity. This is not a sample-related effect, but is due to intrinsic changes in the pumping laser intensity.

In a similar manner, we determined *t*_req_ for CPA_2_PbCl_4_. To this end, we collected TR-SHG traces for the **I** → **II** PT at four different d*T*/d*t* rates, as depicted in Figure 10a. As expected for a first-order PT, an increase in the heating–cooling rate increases temperature hysteresis. The determined Δ*T* values are 2.2, 7.0, 10.3, and 15 K for 5, 10, 20, and 50 K/min d*T*/d*t* rates, giving rise to *t*_req_ values of 26, 42, 32, and 18 s, respectively. Although all these values are essentially under 1 min, it is apparent that the longest transition time is not at the slowest ramp but at 10 K/min, indicating the highest kinetic barriers at this rate. It should be noted that, effectively, the fastest crystal phase switching occurs at 50 K/min with a *t*_req_ of 18 s. Counterintuitively, the second fastest *t*_req_ value is found for a rate of 5 K/min. This, in turn, underlines that the heating–cooling speed alone can be a misleading descriptor for SHG switching performance, although generally a higher d*T*/d*t* tends to correlate with a lower *t*_req_ parameter.

With the *t*_req_ results, we proceeded to a conventional SHG switching study. To do this, we tracked the SHG signal evolution at d*T*/d*t* rates of 5, 10, 20, and 50 K/min while cycling between temperature points corresponding to the completely converted crystal phases **I** and **II** of CPA_2_PbCl_4_. Figure 10b presents the temperature plot of SHG intensities collected at a 50 K/min rate, with plots for the other d*T*/d*t* rates available in Figure S18. Figure 10b demonstrates that the SHG intensity remains consistent for the SHG-*on* state after 18 cycles of switching; similar observations were made for the slower rates of 5, 10, and 20 K/min, each collected over 10 SHG-*on*–SHG-*off* cycles. Since all switching measurements were conducted on the same CPA_2_PbCl_4_ sample, it can be concluded that the switching functionality remains intact after a total of ca. 50 switching cycles. Additionally, using the cycling data, we calculated the SHG contrast ratio. Here, we define the contrast ratio as the integral area of the SHG signal within the 650–750 nm spectral range divided by the integral area of the background (noise) signal of the SHG-*off* state, integrated in the same range. In this way, we yield a value of ca. 300 to 1. This high value, along with the material’s good relative SHG efficiency, underscores the high SHG brightness of the studied material.

## Conclusions

Amine halogenation, or halogen engineering, has emerged as a potent approach for synthesizing hybrid perovskite materials with noncentrosymmetric structures and pronounced polar distortions, quite often culminating in ferroelectric property. In this vein, we employed a chlorinated amine (3-chloropropylamine, CPA), whose 2:1 compound with lead chloride yielded a novel layered hybrid organic–inorganic perovskite, CPA_2_PbCl_4_. The defining structural feature of this material is the richness of its temperature-dependent phase behavior. Indeed, DSC data reveal a diverse polymorphic landscape of this compound, with a total of four identified phases. SCXRD studies show that the HT phase **I** is centrosymmetric, undergoing sequential phase transitions upon cooling at *T*_1_ = 343.9 K, *T*_2_ = 208.6 K, and *T*_3_ = 178.2 K, resulting in polar phases characterized by *Cmc*2_1_ (phase **II**), *Pna*2_1_ (phase **III**), and *Pca*2_1_ (phase **IV**) space groups, respectively. The multi-noncentrosymmetric nature of phases **II**, **III**, and **IV** is corroborated by observation of the SHG activity in each of these three phases, with a general trend of increasing SHG activity with lowering temperatures.

Structural studies also demonstrate that the **I** to **II** PT is related to the *mmmFmm*2 symmetry reduction and is ferroelectric, i.e., without the formation of ferroelastic strain. Ferroelectricity of phase **II** is confirmed by the pyroelectric effect, showing a reversible pyroelectric current upon reversing the poling filed. The in-plane spontaneous polarization along the *c* direction develops in all phases stable below *T*_1_, with the primary component of this polarization arising from the internal dipoles of the molecular CPA^+^ counterions. Interestingly, the structural changes result in linear negative thermal expansion in the *b* direction, and further extension of the lattice parameter *b* is observed when the structure transforms to phases **III** and **IV**.

Thermal, structural, dielectric, and Raman studies also show that the **I** to **II** PT has an order–disorder character, whereas the two remaining PTs are associated with the rearrangement of CPA^+^ cations and distortion of the inorganic layers. Dielectric data reveal the presence of the dielectric relaxation process with the activation energies of 0.07, 0.19, and 0.19 eV in phases **IV**, **III**, and **II**, respectively, as well as ionic conductivity with the activation energies of 0.95 eV (phase **II**) and 1.1 eV (phase **I**).

Linear optical studies revealed that CPA_2_PbCl_4_ exhibits broadband emission with two bands located at 80 K near 390 and 598 nm. The higher energy band shows a pronounced redshift on heating, leading to significant thermochromism of the observed PL. As a result, the color of the observed PL changes from orange-yellow at 80 K to white above 180 K.

Finally, the HT SHG-*on*–SHG-*off* functionality of CPA_2_PbCl_4_ has been demonstrated by a set of rate-dependent (5–50 K/min) temperature cycling experiments, which demonstrated high switching stability and a high SHG contrast ratio of 300:1. Even more importantly, these data allowed us to employ for analysis a recently introduced metric by our team, the time requirement parameter (*t*_req_), which provides a means of comparing the duration of switching events. We calculated a *t*_req_ value of 18 s for a 50 K/min rate and found that the slowest rate of temperature change does not necessarily result in the slowest phase switching.

A pertinent question arising from these results is how fast SHG switching can actually be considered *fast*. Given the current lack of kinetic data for SHG switching studies, we refrain from answering this question definitively for now. To provide a well-informed answer, the SHG switching community should increasingly adopt objective metrics, such as *t*_req_, to enable quantitative comparisons between materials. However, some rough estimates can be made. For instance, we expect that for most real-life applications, a *t*_req_ of several dozen seconds is likely too long. A potential milestone for the SHG field could be achieving *t*_req_ in the range of single seconds, which would require materials with very narrow thermal hysteresis, such as those undergoing reversible structural transformations with minimal energy barriers. This suggests that, for example, materials with extensive hydrogen bonding are unlikely to show unusually rapid SHG switching times.

## Experimental Section

### Synthesis

Single crystals of CPA_2_PbCl_4_ were grown using the slow evaporation method. In this method, 3 mmol of PbCl_2_ (98%, Sigma-Aldrich) and 6 mmol of 3-chloropropylamine hydrochloride (98%, Sigma-Aldrich) were dissolved in *N*,*N*-dimethylformamide under stirring on a hot plate at 40 °C. The solution was filtered and left undisturbed at room temperature (RT). The colorless, elongated, plate-like crystals with dimensions up to 3 mm, which grew in the reaction vial, were separated from the liquid after 4 days and dried at RT. The comparison of the powder XRD pattern of CPA_2_PbCl_4_ with the calculated one based on the single-crystal data attests to the phase purity of bulk sample (Figure S19).

### Powder X-ray Diffraction

Powder X-ray diffraction (PXRD) patterns of the ground crystals were measured in the reflection mode using an X’Pert PRO X-ray diffraction system equipped with a PIXcel ultrafast line detector and Soller slits for CuK_α1_ radiation (λ = 1.54056 Å).

### DSC Measurements

Calorimetry measurements were performed using a Mettler Toledo DSC-3 calorimeter in the temperature range of 120–380 K. Nitrogen was used as a purging gas, and the heating and cooling rates were 5 K/min. The sample weight was 20.37 mg. The excess heat capacity associated with the PT was calculated by subtracting the baseline data, representing the system variation in the absence of the PTs.

### Single-Crystal X-ray Diffraction

Single-crystal X-ray diffraction (SCXRD) experiments were conducted using an Oxford Diffraction (OD) Xcalibur four-circle diffractometer with an Atlas CCD detector and utilizing graphite-monochromated MoK_α_ radiation (λ = 0.7107 Å). The temperature was controlled by a nitrogen open-flow system (Cryostream 1000, Oxford Cryosystems). Absorption was corrected via multiscan methods using CrysAlis PRO (Rigaku OD, 2023). Crystal structures were solved in Olex2 1.5 using SHELXT and refined with the SHELXL program.^50−52^ Empirical absorption correction was applied with spherical harmonics implemented in the SCALE3 ABSPACK scaling algorithm. In the high-temperature (HT) phase **I**, hydrogen atoms were not included into refinement due to the disorder. In phases **II**–**IV**, hydrogen atoms were placed at calculated positions and refined as riding atoms. Crystal data, data collection, and refinement results of the subsequent phases are summarized in Table S1. The main geometric details are provided in Tables S2 and S3.

The brief structural data of CPA_2_PbCl_4_: (**I**, 360 K): orthorhombic, *Cmce*, *a* = 28.552(7) Å, *b* = 7.855(3) Å, *c* = 7.831(3) Å, *V* = 1756.4(10) Å^3^, *Z* = 4, *R*_1_ = 0.039, *wR*_2_ = 0.110, *S* = 1.03; (**II**, 295 K): orthorhombic, *Cmc*2_1_, *a* = 26.725(7) Å, *b* = 8.019(3) Å, *c* = 7.812(3) Å, *V* = 1674.1(10) Å^3^, *Z* = 4, *R*_1_ = 0.030, *wR*_2_ = 0.135, *S* = 1.04; (**III**, 190 K): orthorhombic, *Pna*2_1_, *a* = 16.222(5) Å, *b* = 26.432(7) Å, *c* = 7.639(3) Å, *V* = 3275.4(19) Å^3^, *Z* = 8, *R*_1_ = 0.030, *wR*_2_ = 0.135, *S* = 1.00; (**IV**, 100 K): orthorhombic, *Pca*2_1_, *a* = 16.307(5) Å, *b* = 26.293(7) Å, *c* = 7.537(3) Å, *V* = 3231.5(18) Å^3^, *Z* = 8, *R*_1_ = 0.025, *wR*_2_ = 0.047, *S* = 1.19.

### Raman Spectroscopy

Temperature-dependent Raman spectra in the 3500–50 cm^–1^ range were measured using a Renishaw inVia Raman spectrometer equipped with a confocal DM2500 Leica optical microscope and a CCD detector. The 488 nm line of an argon laser was used as the excitation source. The second experiment was performed in the 250–10 cm^–1^ range using the same spectrometer and an Eclipse filter. The temperature of the sample was controlled using a THMS600 temperature control stage, and the spectral resolution was 2 cm^–1^.

### Broadband Dielectric Spectroscopy (BDS) and Pyroelectric Current Measurement

Dielectric measurements were carried out using a Broadband Impedance Novocontrol Alpha-A analyzer. A sinusoidal voltage with an amplitude of 1 V and frequency in the range of 1–10^6^ Hz was applied across the sample. The samples were measured on a polycrystalline pallet with a diameter of 5 mm and a thickness of 0.54 mm. To ensure proper electrical contact, silver paste was applied to the parallel surfaces of the samples. The temperature was controlled by the Novocontrol Quattro system using a nitrogen gas cryostat. The measurement was made in the temperature range from 130 to 380 K with a temperature stability of better than 0.1 K. Before measurements, the sample was heated for 15 min at a temperature of 350 K. A pyroelectric current measurement was performed on a single crystal along the *c* polar direction with silver electrical contacts. Current measurements were carried out using a Keithley 6514 electrometer during heating of the sample, with a rate of 2 K/min.

### Linear Optical Studies

The RT absorption spectra of the powdered samples were measured using a Varian Cary 5E UV–vis-NIR spectrophotometer (Varian). Temperature-dependent emission spectra were recorded under 266 nm excitation from a laser diode and with a Hamamatsu photonic multichannel analyzer PMA-12, equipped with a BT-CCD linear image sensor (Hamamatsu Photonics). The temperature of the samples was controlled by using a Linkam THMS 600 heating/freezing stage (Linkam).

### SHG Studies

Nonlinear optical (NLO) experiments were performed using a laser system employing a wavelength-tunable Light Conversion Topaz Prime Vis-NIR optical parametric amplifier (OPA) pumped by a Coherent Astrella Ti:sapphire regenerative amplifier providing femtosecond laser pulses (800 nm, 75 fs) at a 1 kHz repetition rate. The output of OPA was set to 1400 nm, and the laser fluence at samples was equal to 0.19 mJ/cm^2^. The single crystals of CPA_2_PbCl_4_ and KDP were crushed with a spatula and sieved through an Aldrich mini-sieve set, collecting a microcrystal size fraction of 88–125 μm. Next, size-graded samples were fixed between microscope glass slides to form tightly packed layers, sealed, and mounted to the horizontally aligned sample holder. No refractive index matching oil was used. The employed measurement setup operates in the reflection mode; a schematic diagram of the setup is shown in Figure S20. Specifically, the laser beam was directed onto the sample at 45° to its surface. Emission collecting optics consisted of a Ø25.0 mm plano-convex lens of focal length 25.4 mm mounted to the 400 μm 0.22 NA glass optical fiber and was placed along the normal to the sample surface. The distance between the collection lens and the sample was equal to 30 mm. The spectra of the temperature-dependent NLO responses were recorded by an Ocean Optics Flame T XR fiber-coupled CCD spectrograph with a 200 μm entrance slit. Scattered pumping radiation was suppressed with the use of a Thorlabs 750 nm hard-coated short-pass dielectric filter. The temperature control of the sample was performed by using a Linkam LTS420 heating/freezing stage. Temperature stability was equal to 0.1 K. TR-SHG study of CPA_2_PbCl_4_ was conducted in a range of 178–373 K. A Kurtz–Perry (Graja) powder test^53,54^ was performed by comparing the SHG signal of CPA_2_PbCl_4_ with that of the KDP standard at 293 K. The SHG switching experiment was conducted by cycling the sample’s temperature at rates of 5, 10, 20, and 50 K/min within the ranges of 335–360 K (10 cycles), 335–360 K (10 cycles), 332–357 K (10 cycles), and 330–360 K (18 cycles), respectively. It is important to note that the measurement window for the 50 K/min rate is broader by 5 K due to larger thermal hysteresis. To enhance the visibility of the individual switching cycles, isothermal periods of 30, 30, 15, and 15 s for rates of 5, 10, 20, and 50 K/min, respectively, were employed once the highest temperature, corresponding to the SHG-*off* state, was reached. The same laser setup and geometry was used for all SHG experiments. The same sample was used for all the SHG switching experiments.

## Supporting Information Available

Tables S1–S4: crystallographic data and Raman wavenumbers together with assignments; Figures S1–S20: powder X-ray diffraction patterns, DSC traces, thermal evolution of lattice parameters, crystal structures, Raman, dielectric, SHG, absorption and PL spectra, temperature dependence of integrated emission intensity and PL band positions, plots of SHG intensities obtained for temperature switching experiments; and schematic diagram of the optical setup used for SHG studies (PDF)
Structures at 360 K (CIF)
Structures at 295 K (CIF)
Structures at 190 K (CIF)
Structures at 100 K (CIF)

## Supplementary Material

am4c14244_si_001.pdf

am4c14244_si_002.cif

am4c14244_si_003.cif

am4c14244_si_004.cif

am4c14244_si_005.cif
