# Supplementary material for: Multi-Noncentrosymmetric Polar Order in 2D Hybrid Lead Chloride with Broadband Emission and High-Temperature Second-Harmonic Generation Switching
Source: ACS Appl Mater Interfaces. 2024 Oct 24;16(44):60564–75. doi: 10.1021/acsami.4c14244 (PMC11551948; doi:10.1021/acsami.4c14244)
Supplement: Supplementary file 1 — am4c14244_si_001.pdf [file am4c14244_si_001.pdf]

Supporting Information for

Multi-Noncentrosymmetric Polar Order in 2D Hybrid Lead Chloride with Broadband Emission and High-Temperature Second Harmonic Generation Switching

Mirosław Mączka, [†,*] Jan K. Zaręba,[*,‡] Anna Gągor, [†] Katarzyna Fedoruk-Piskorska, [‖] Dagmara Stefańska, [†] Dawid Drozdowski, [†] Maciej Ptak, [†] and Adam Sieradzki [‖]

[†] W. Trzebiatowski Institute of Low Temperature and Structural Research of the Polish Academy of Sciences, Okólna 2, 50-422 Wroclaw, Poland, E-mail: m.maczka@intibs.pl

[‡] Institute of Advanced Materials, Faculty of Chemistry, Wroclaw University of Science and Technology, Wybrzeże Wyspiańskiego 27, 50-370, Wrocław, Poland, E-mail: jan.zareba@pwr.edu.pl

[‖] Department of Experimental Physics, Wrocław University of Science and Technology, Wybrzeże Wyspiańskiego 27, 50-370 Wrocław, Poland

DSC

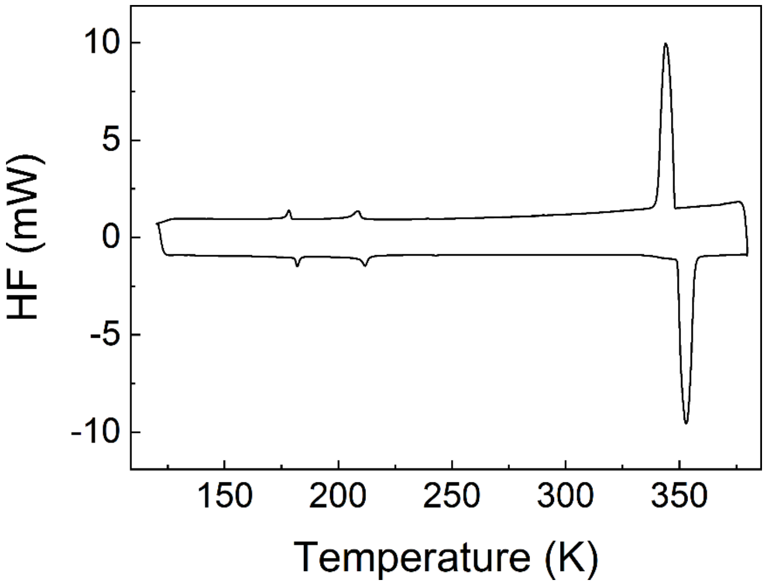

Figure S1. DSC traces for CPA_2PbCl_4 in heating and cooling modes.

SCXRD

Table S1. Experimental and refinement details of CPA_2PbCl_4 (M_r=538.11).

	**I, 360 K**	**II, 295 K**	**III, 190 K**	**IV, 100 K**
**Crystal data**				
Crystal system, space group	Orthorhombic, *Cmce*	Orthorhombic, $Cmc2_1$	Orthorhombic, $Pna2_1$	Orthorhombic, $Pca2_1$
Temperature (K)	360	295	190	100
a, b, c (Å)	28.552(7), 7.855(3), 7.831(3)	26.725(7), 8.019(3), 7.812(3)	16.222(5), 26.432(7), 7.639(3)	16.307(5), 26.293(7), 7.537(3)
α, β, γ (°)	90, 90, 90	90, 90, 90	90, 90, 90	90, 90, 90
V (Å^3)	1756.4(10)	1674.1(10)	3275.4(19)	3231.5(18)
Z	4	4	8	8
μ (mm^{-1})	10.49	11.01	11.26	11.41
Crystal size (mm)	0.36 × 0.15 × 0.05			
**Data collection**				
No. of measured, independent and observed [$I > 2\sigma(I)$] reflections	2373, 916, 512	8141, 1733, 1643	64942, 6698, 5505	63949, 6615, 5768
R_{int}	0.026	0.028	0.034	0.035
**Refinement**				
$R[F^2 > 2\sigma(F^2)]$, $wR(F^2)$, S	0.039, 0.110, 1.03	0.032, 0.084, 1.04	0.030, 0.135, 1.00	0.025, 0.047, 1.19
No. of reflections	916	1733	6698	6615
No. of parameters	34	75	275	275
No. of restraints	5	21	19	1
$\Delta\rho_{max}$, $\Delta\rho_{min}$ (eÅ^{-3})	0.42, -0.95	1.09, -0.72	1.33, -1.21	0.99, -1.17
Absolute structure parameter	-	-0.009 (19)	-0.023 (3)	-0.015 (2)

Table S2. Selected bond lengths in CPA_2PbCl_4. The distances between the Cl atom of CPA^+ and the nearest Cl from the inorganic part are marked in bold.

**I, 360 K**			
Pb1—Cl1^{i}	2.860 (3)	Pb1—Cl1	2.860 (3)
Pb1—Cl1^{ii}	2.860 (3)	Pb1—Cl2^{ii}	2.833 (7)
Pb1—Cl1^{iii}	2.860 (3)	Pb1—Cl2	2.833 (7)
**Cl—Cl**	**5.07**		
**II, 295 K**			
Pb1—Cl1	2.846 (7)	Pb1—Cl3	2.858 (4)
Pb1—Cl1^{iv}	2.875 (5)	Pb1—Cl2	2.926 (5)
Pb1—Cl3^{v}	2.858 (4)	Pb1—Cl2^{vi}	2.943 (6)
**Cl—Cl**	**3.63**		
**III, 190 K**			
Pb1—Cl6^{vii}	2.961 (4)	Pb2—Cl6	2.924 (4)
Pb1—Cl5	2.904 (4)	Pb2—Cl5	2.812 (4)
Pb1—Cl1	2.924 (4)	Pb2—Cl1^{viii}	2.961 (4)
Pb1—Cl4	2.879 (4)	Pb2—Cl7	2.797 (3)
Pb1—Cl2	2.826 (4)	Pb2—Cl2^{ix}	2.897 (4)
Pb1—Cl3	2.830 (3)	Pb2—Cl8	2.919 (3)
**Cl—Cl**	**3.38–3.82**		
**IV, 100 K**			
Pb1—Cl1^{x}	2.972 (2)	Pb2—Cl6	2.909 (2)
Pb1—Cl1	2.914 (2)	Pb2—Cl6^{xii}	2.958 (2)
Pb1—Cl2	2.906 (2)	Pb2—Cl7	2.8120 (16)
Pb1—Cl2^{xi}	2.817 (2)	Pb2—Cl8	2.9015 (18)
Pb1—Cl3	2.7790 (17)	Pb2—Cl5	2.916 (2)
Pb1—Cl4	2.9379 (18)	Pb2—Cl5^{xiii}	2.817 (2)
**Cl—Cl**	**3.30–3.77**		

Symmetry code(s): (i) *x*, *y*-1/2, -*z*+1/2; (ii) -*x*, -*y*, -*z*; (iii) -*x*, -*y*+1/2, *z*-1/2; (iv) -*x*, -*y*+2, *z*-1/2; (v) -*x*, *y*, *z*; (vi) -*x*, -*y*+1, *z*-1/2; (vii) *x*+1/2, -*y*+1/2, *z*; (viii) *x*-1/2, -*y*+1/2, *z*-1; (ix) *x*, *y*, *z*-1; (x) -*x*+1, -*y*, *z*+1/2; (xi) -*x*+3/2, *y*, *z*-1/2; (xii) -*x*+3/2, *y*, *z*+1/2; (xiii) -*x*+1, -*y*+1, *z*-1/2.

Table S3. Selected hydrogen-bond parameters of CPA_2PbCl_4.

D*—H···*A	***D*—H (Å)**	**H···*A* (Å)**	***D*···*A* (Å)**	***D*—H···*A* (°)**
**II, 295 K**				
N1—H1A···Cl3^{i}	0.89	2.39	3.238 (14)	159.0
N1—H1B···Cl3	0.89	2.34	3.226 (14)	171.2
N1—H1C···Cl1^{ii}	0.89	2.61	3.312 (14)	136.3
N1—H1C···Cl2	0.89	2.82	3.338 (12)	118.2
**III, 190 K**				
N9—H9A···Cl4	0.91	2.28	3.186 (11)	170.7
N9—H9B···Cl7^{iii}	0.91	2.34	3.242 (12)	172.8
N9—H9C···Cl6^{iii}	0.91	2.70	3.305 (11)	124.7
N9—H9C···Cl2	0.91	2.62	3.306 (12)	132.8
N5—H5A···Cl7	0.91	2.34	3.243 (12)	172.2
N5—H5B···Cl4^{iv}	0.91	2.31	3.182 (12)	160.6
N5—H5C···Cl5	0.91	2.64	3.363 (12)	136.9
N5—H5C···Cl1^{iv}	0.91	2.81	3.348 (11)	118.8
N13—H13A···Cl8	0.91	2.29	3.190 (11)	169.1
N13—H13B···Cl5	0.91	2.59	3.313 (11)	136.6
N13—H13B···Cl1^{iv}	0.91	2.72	3.294 (11)	121.8
N13—H13C···Cl3^{iv}	0.91	2.33	3.234 (11)	174.7
N1—H1A···Cl3	0.91	2.30	3.203 (11)	175.1
N1—H1B···Cl6^{iii}	0.91	2.74	3.359 (11)	126.4
N1—H1B···Cl2	0.91	2.69	3.354 (12)	130.6
N1—H1C···Cl8^{iii}	0.91	2.30	3.169 (11)	160.7
**IV, 100 K**				
N5—H5A···Cl1^{v}	0.91	2.63	3.325 (6)	134.3
N5—H5A···Cl2	0.91	2.77	3.373 (6)	124.8
N5—H5B···Cl3^{vi}	0.91	2.34	3.239 (6)	169.9
N5—H5C···Cl4^{v}	0.91	2.29	3.166 (6)	160.1
N1—H1A···Cl1	0.91	2.63	3.279 (6)	128.5
N1—H1A···Cl2^{vii}	0.91	2.62	3.287 (6)	130.7
N1—H1B···Cl4^{viii}	0.91	2.27	3.177 (6)	176.9
N1—H1C···Cl3	0.91	2.35	3.254 (6)	171.7
N13—H13A···Cl6	0.91	2.80	3.354 (6)	120.3
N13—H13A···Cl5^{ix}	0.91	2.62	3.354 (6)	138.3
N13—H13B···Cl7	0.91	2.35	3.219 (6)	160.0
N13—H13C···Cl7^{vi}	0.91	2.35	3.251 (7)	170.2
N9—H9A···Cl8^{x}	0.91	2.30	3.205 (6)	171.4
N9—H9B···Cl8^{xi}	0.91	2.24	3.151 (6)	175.3
N9—H9C···Cl6^{x}	0.91	2.64	3.272 (6)	127.3
N9—H9C···Cl5	0.91	2.60	3.271 (6)	131.0

Symmetry code(s): (i) *x*, -*y*+1, *z*-1/2; (ii) -*x*, -*y*+1, *z*-1/2; (iii) *x*+1/2, -*y*+1/2, *z*+1; (iv) *x*-1/2, -*y*+1/2, *z*; (v) *x*+1/2, -*y*, *z*; (vi) -*x*+3/2, *y*, *z*+1/2; (vii) *x*-1/2, -*y*, *z*; (viii) -*x*+1, -*y*, *z*+1/2; (ix) *x*+1/2, -*y*+1, *z*; (x) *x*-1/2, -*y*+1, *z*; (xi) -*x*+1, -*y*+1, *z*+1/2.

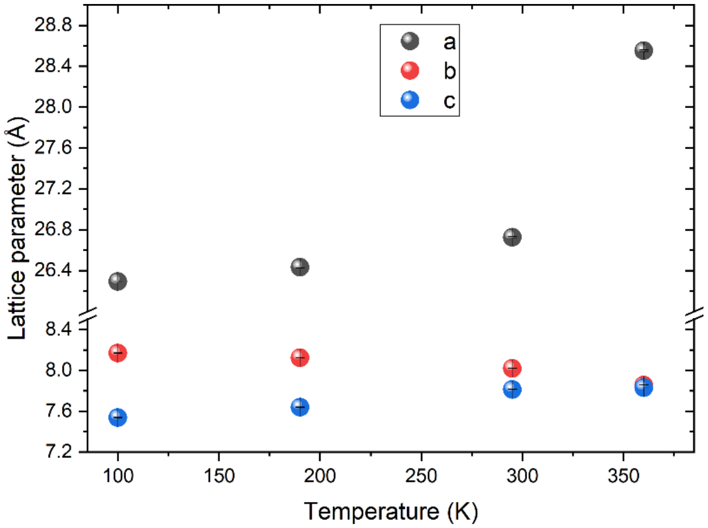

Figure S2. Lattice parameters in CPA_2PbCl_4.

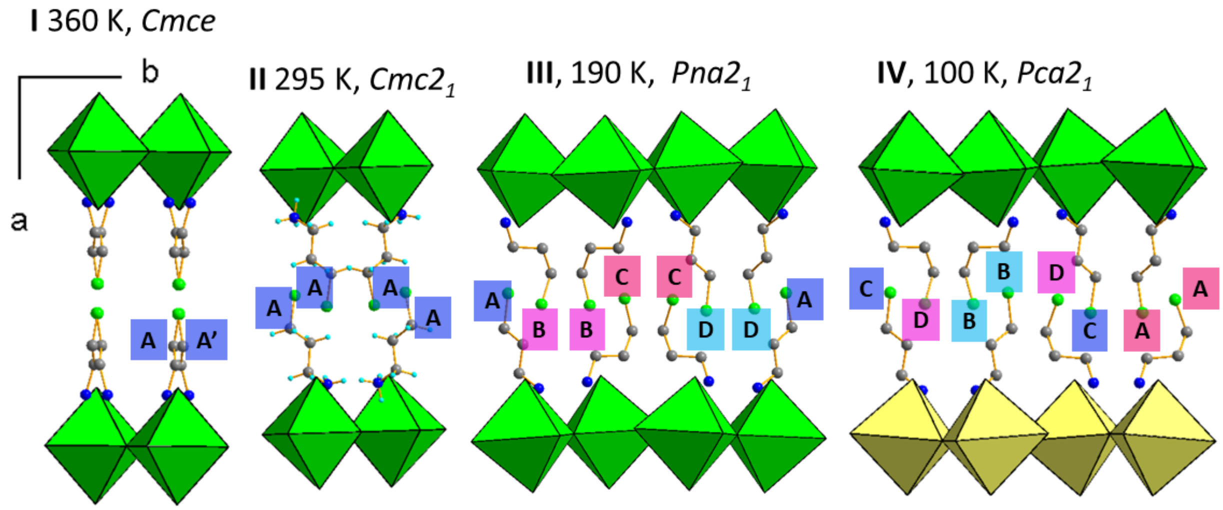

Figure S3. Structure transformations in subsequent phase transitions in CPA_2PbCl_4.

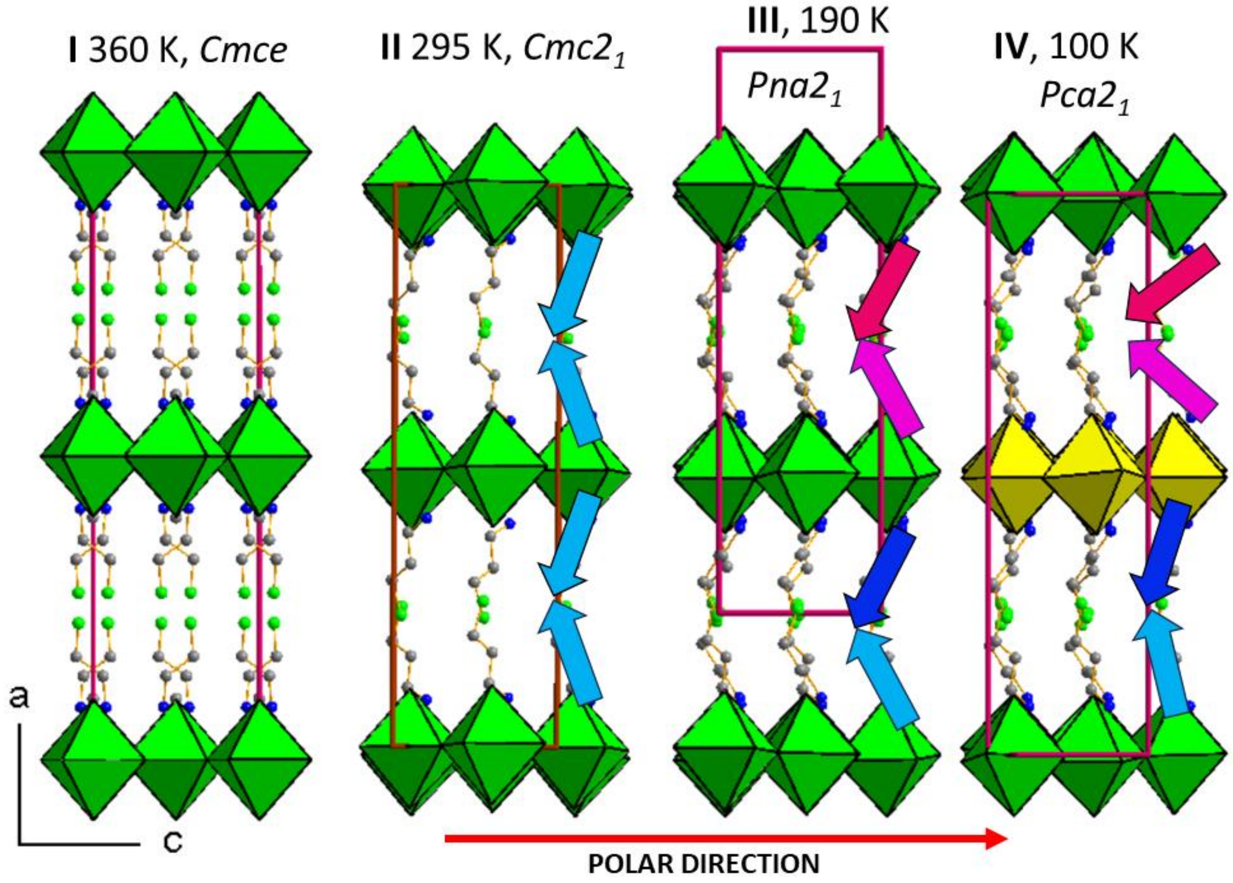

Figure S4. The development of the spontaneous polarization in LT phases of CPA_2PbCl_4.

Raman

Table S4. Raman wavenumbers (in cm^{-1}) of CPA_2PbCl_4 at 360, 220, 180 and 80 K together with the proposed assignment.[a]

360 K (*Cmce* phase)	210 K (*Cmc*2_1 phase)	180 K (*Pna*2_1)	80 K (*Pca*2_1)	assignment
3190m,b	3211m	3212m	3218m+3214m+3205w	$\nu_{as}(NH_3)$
3122m,b				$\nu_{as}(NH_3)$
3007w	3007w+2998w	3010w+2998w	3013w+3007w+2998w+2995w	$\nu_{as}(CH_2)$
2967m	2976sh+2970m	2979sh+2970m	2985w+2976w+2970m	$\nu_s(CH_2)$
2951sh	2960w	2960w+2954sh	2963sh+2960w+2955w	$\nu_s(CH_2)$
	2932w	2944vw+2933w	2948w+2941vw+2935w	$\nu_s(CH_2)$
2922m	2913m+2907sh	2916sh+2911m+2907m	2918m+2909m+2905m	$\nu_s(CH_2)$
2877w	2879w	2888sh+2880w	2888w+2881w	overtone/combination
1598w	1600w	1607vw+1601w	1611vw+1603w	$\delta_{as}(NH_3)$
1577w	1574w	1575w	1573w	$\delta_{as}(NH_3)$
	1507w+1492w	1495w	1510w+1502w+1496w+1490w	$\delta_s(NH_3)$
1477w	1477w+1475sh	1480w	1481w	$\delta(CH_2)$
1448s	1457w+1446s	1459w+1447s	1461m+1447s	$\delta(CH_2)$
1429w	1432w+1423w	1432w+1423w	1431w+1420w	$\delta(CH_2)$
1396w,b	1401w	1401w	1403vw	$\delta(CH_2)$
1358w				$\omega(CH_2)$
1337m	1340s	1340m	1331w+1327w	$\omega(CH_2)$
1325m	1327m	1330m	1307m+1300sh	$\omega(CH_2)$
1266m	1265m	1266m+1262m	1268w+1263m	$\tau(CH_2)$
1259m	1257m	1256m	1256m	$\tau(CH_2)$
1218w	1220m	1221m	1220m	$\tau(CH_2)$
1137vw	1141m	1142m	1145m+1143m	$\rho(NH_3^+)$
1124w	1129w	1131w	1133w+1129w	$\rho(CH_2)$
1098m	1098m+1090m	1103w+1097w+1090m	1103w+1096w+1091w	$\rho(CH_2)$
1043m	1055m+1042w	1055m+1042m	1056m+1042m	$\nu(CN)$

1000w	1009w+999m	1012w+999w	1013w+1001w	ν_{as}(CCC)
971m	974m	980m+971sh	981w+975w	ω(NH,CH)
940s	945s	946s	947s	ν_s(CCC)
913w	931w	932w	933w+929w	overtone
881m				ρ(CH_2)
835m	843m	844m	845w	ρ(CH_2)
826m				ρ(CH_2)
780m	780m+770m	780w+773sh+768m	771m+766m	ρ(CH_2)
	757w	759w	763w	ρ(CH_2)
737m	740s	742s	744m+740s	ν(CCl)
727m	719vw	721vw	725vw+720vw	ρ(CH_2)
657m	657s	657s	657s	ν(CCl)
486w	487w	488w	487w	δ(CCN)
460w				δ(CCN)
429w	430w	431w	431w	δ(CCC)
336m	337s	337s	345w+337s	δ(CCCl)
312w	311w	311w	311w	δ(CCCl)
255sh	270w	272w+266sh	277w+266w	τ(NH_3)
179m	178m	174s+165sh	192m +177vs+168m+161sh	L+ T'+ Pb-Cl stretch
116s	133w	135w	147w+141w	L+ T'+ Pb-Cl stretch
	107s	124m+108s	129m+116m+108vs	L+ T'+ Pb-Cl stretch
88s	94m	97m	103s+99s	Pb-Cl bend
			89vs+84m	Pb-Cl bend
	74vs	76s	77vs	Pb-Cl bend
			72s+69s	Pb-Cl bend
55s	60vs	61s	65m+57m	Pb-Cl bend
		51w	54w+50w	Pb-Cl bend
35vs	42vs	42s	46w+44w+40m	Pb-Cl bend
20vs	20s	21m	21m	L($PbCl_6$)

[a] key: vs, very strong; s, strong; m, medium; w, weak; vw, very weak; sh, shoulder; ν, stretching; δ, bending (scissoring); ρ, rocking; ω, wagging; τ, twist; L, librational mode of organic cation; T', translational mode of organic cation

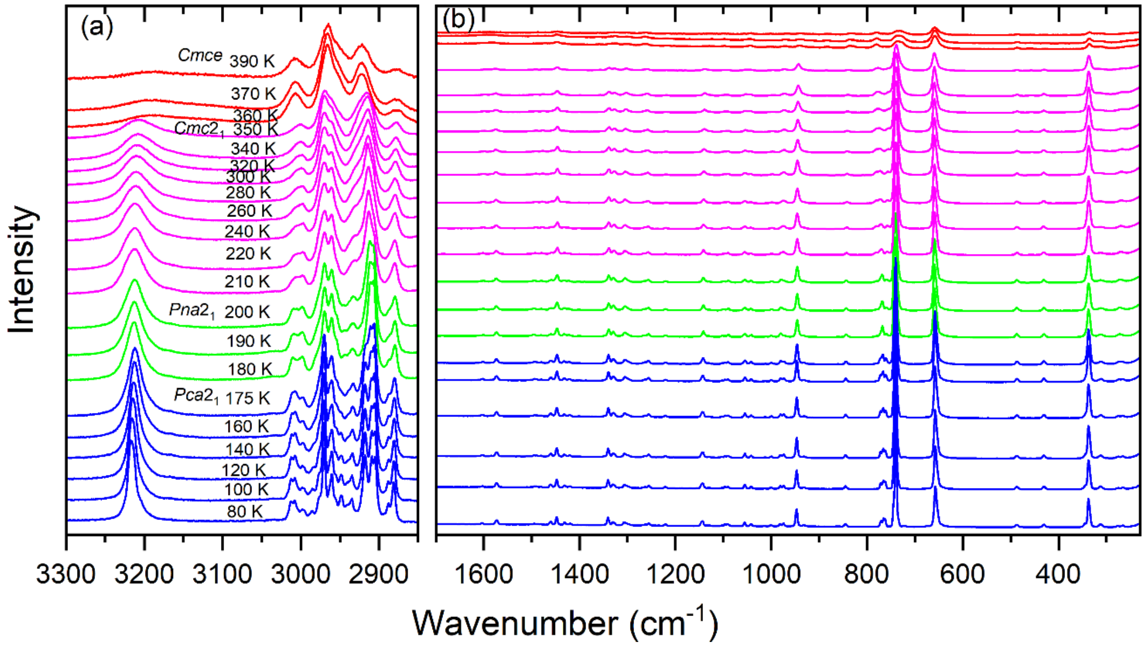

Figure S5. Raman spectra of CPA_2PbCl_4 in (a) 3300–2850 cm^{-1} and (b) 1700–230 cm^{-1} range.

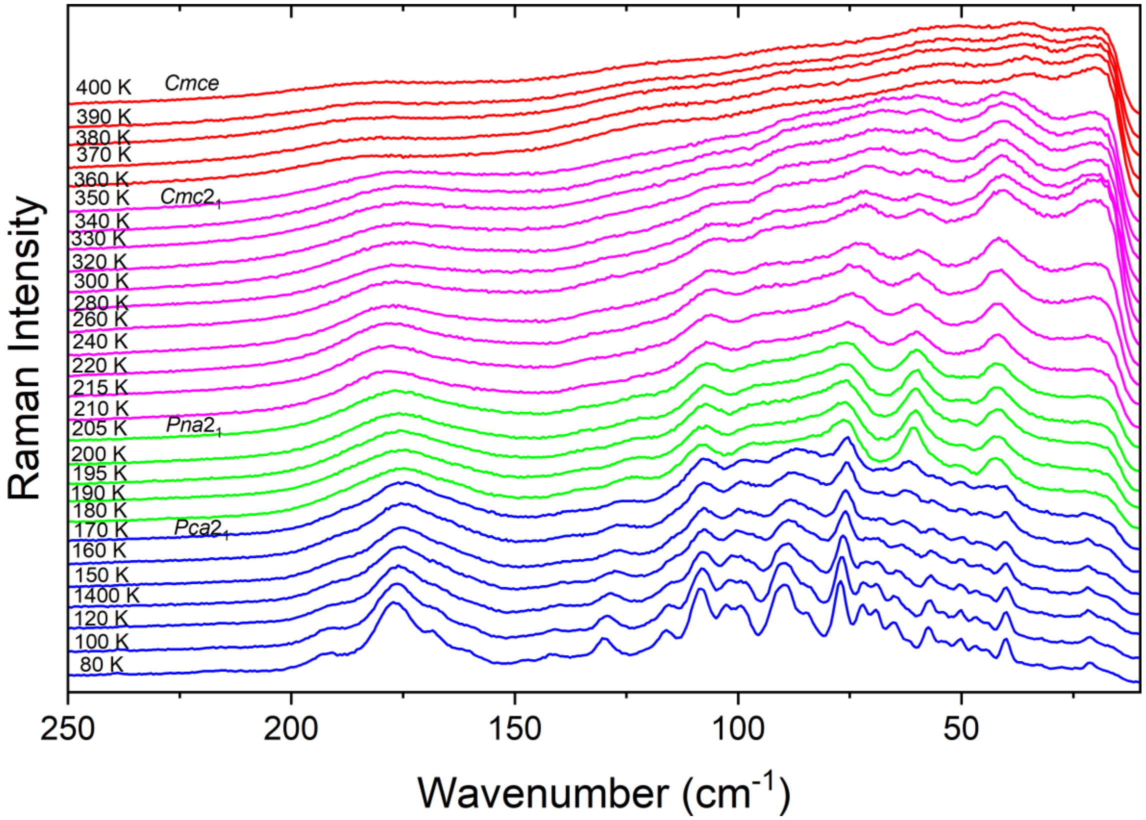

Figure S6. Raman spectra of CPA_2PbCl_4 in 250-10 cm^{-1} range.

BDS

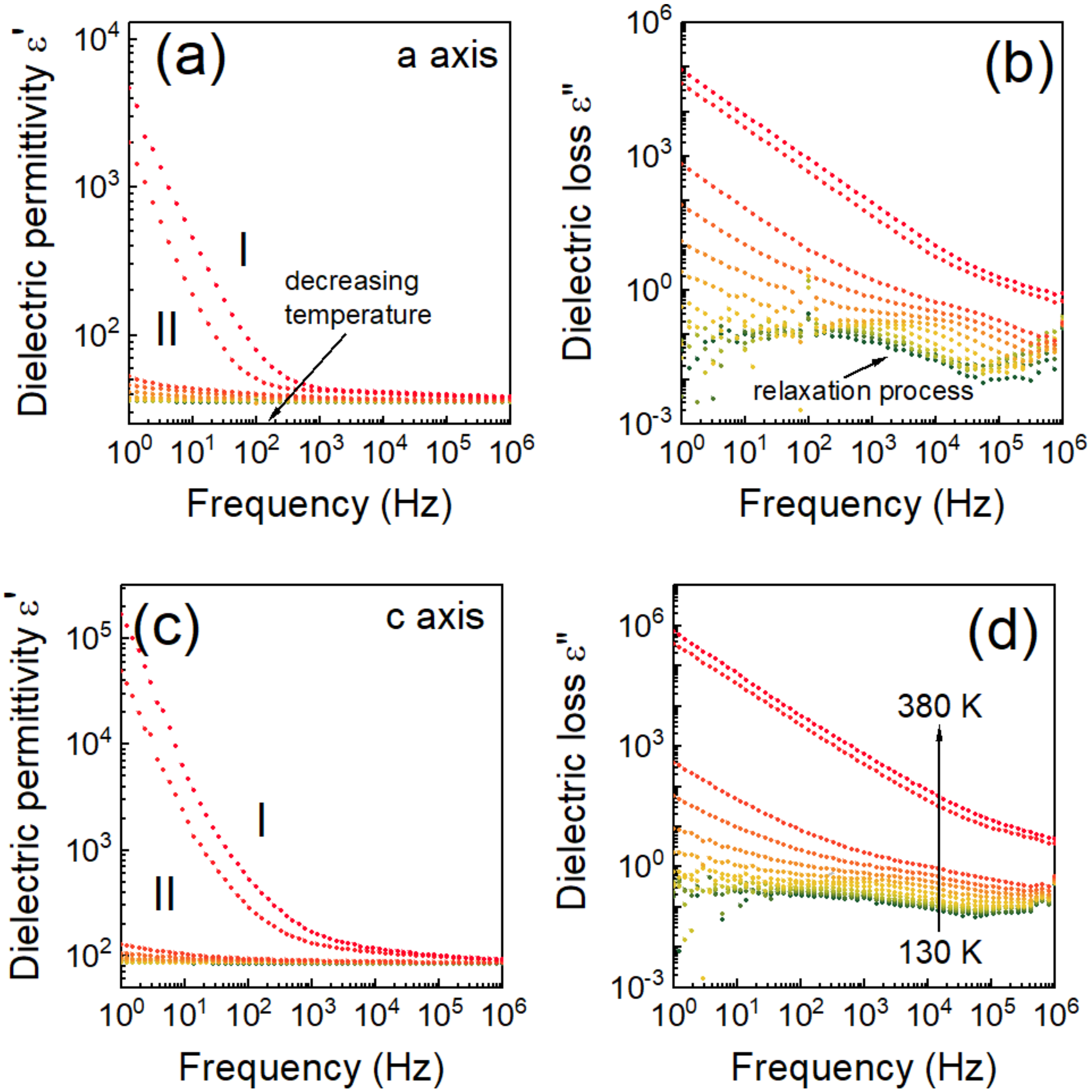

Figure S7. Frequency dependence of complex permittivity measured along the *a* axis (panels a and b) and the *c* axis (panels c and d) in the temperature range from 130 to 380 K for CPA_2PbCl_4.

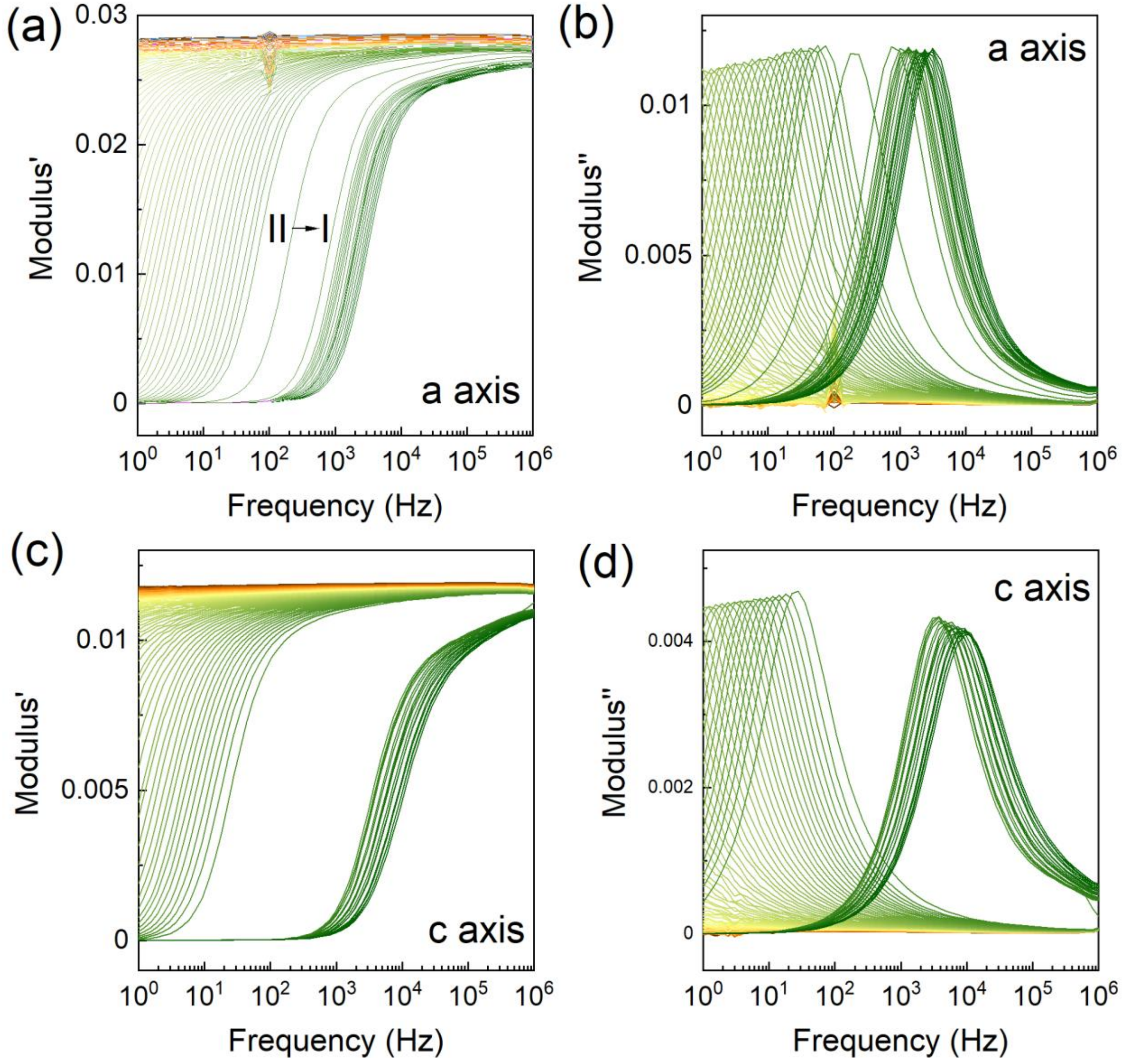

Figure S8. Frequency dependence of complex modulus measured along the *a* axis (panels a and b) and the *c* axis (panels c and d).

Linear optical studies

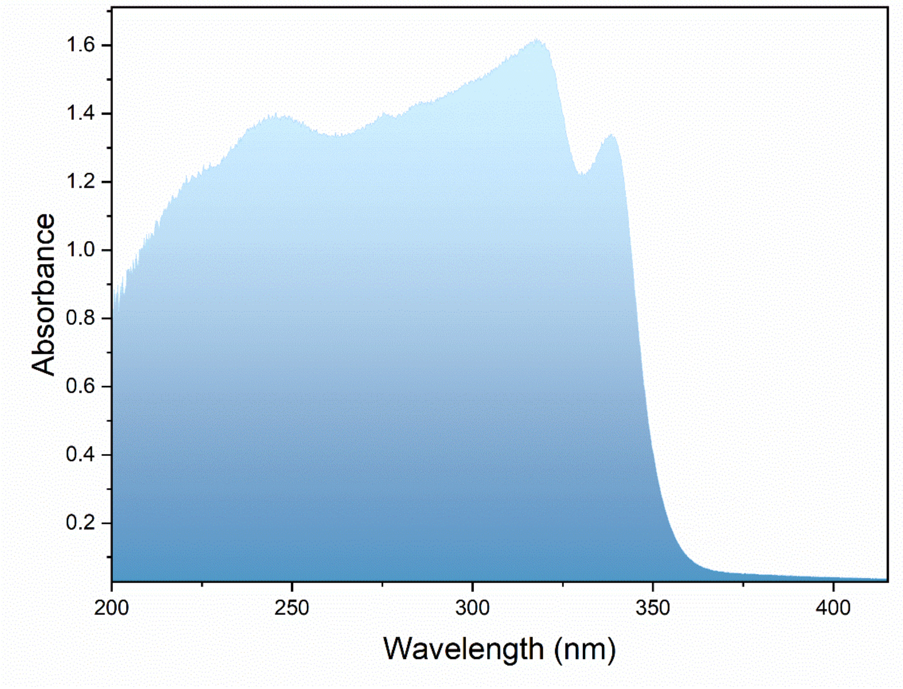

Figure S9. Diffuse absorption spectrum of CPA_2PbCl_4 registered at 300 K.

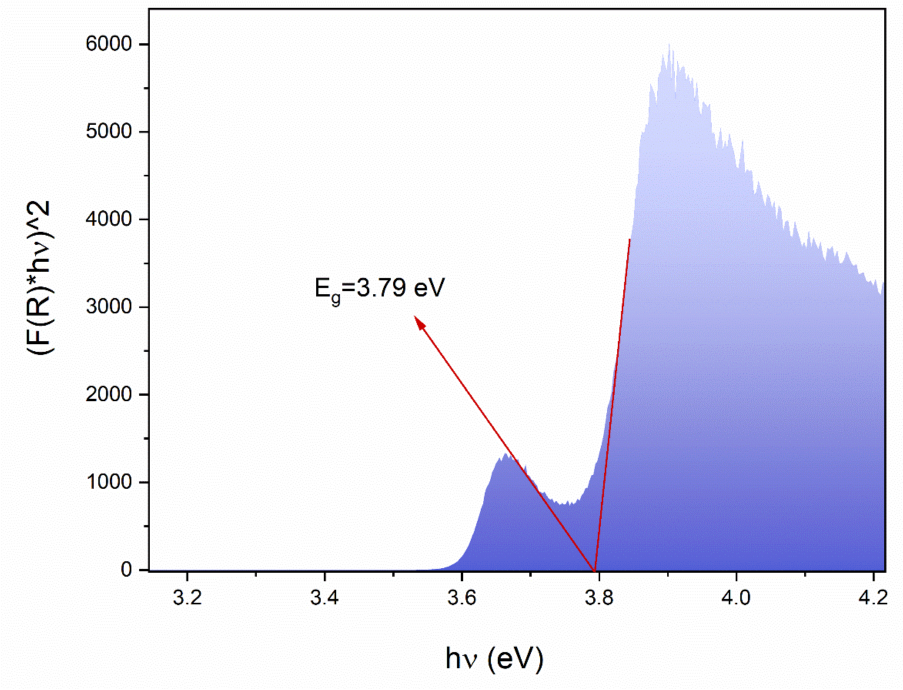

Figure S10. The energy band gap of CPA_2PbCl_4 estimated using the Kubelka-Munk function.

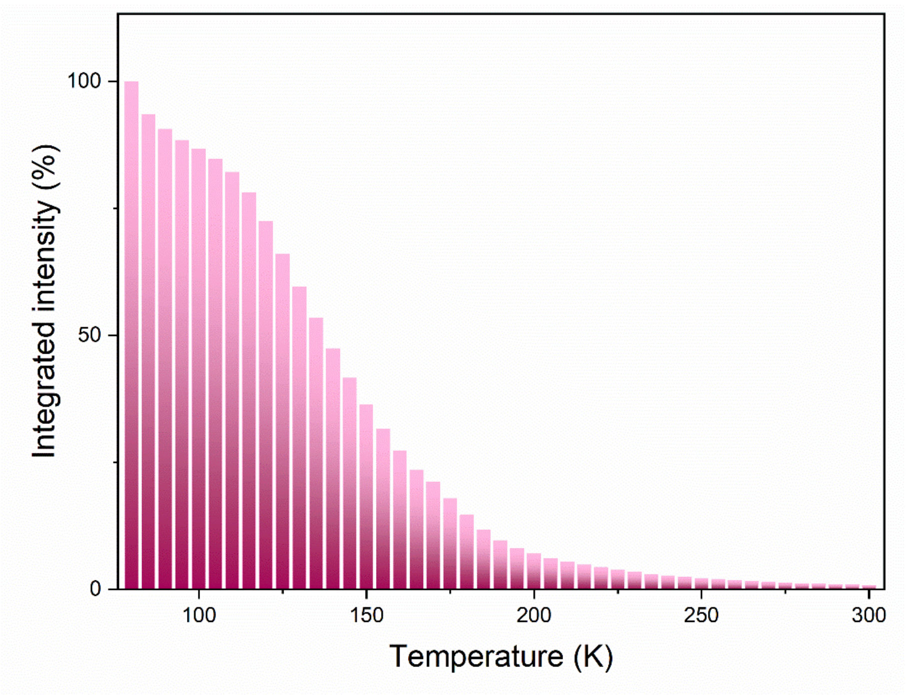

Figure S11. Normalized integrated emission intensity as a function of the temperature of broad emission band, denoted as P_2, of CPA_2PbCl_4.

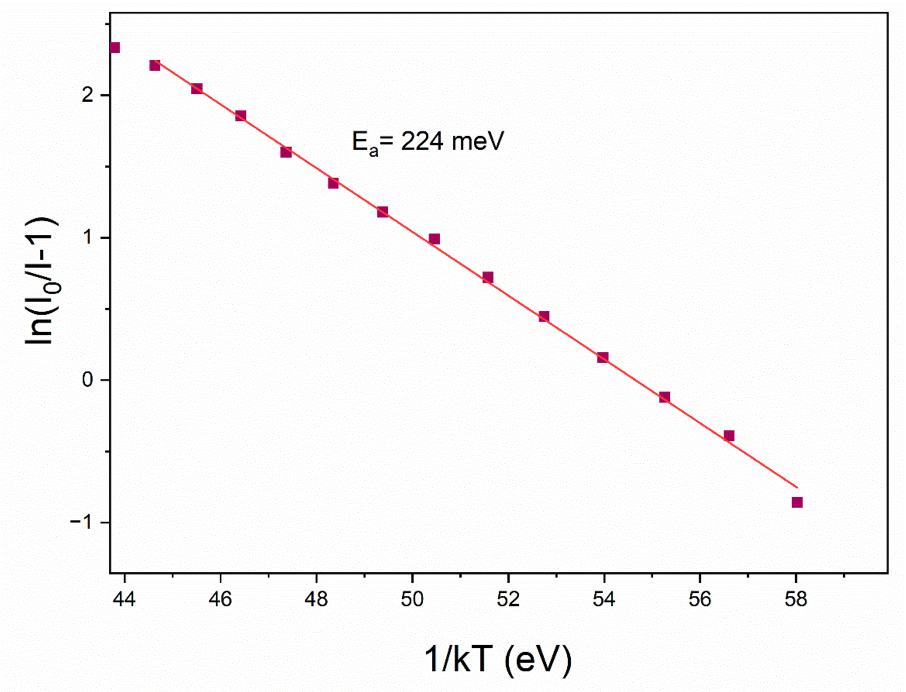

Figure S12. Logarithm of I_0/I-1 as a function of $1/kT$, of broad emission denoted as P_2 of CPA_2PbCl_4. E_a was extracted by fitting the Arrhenius equation.

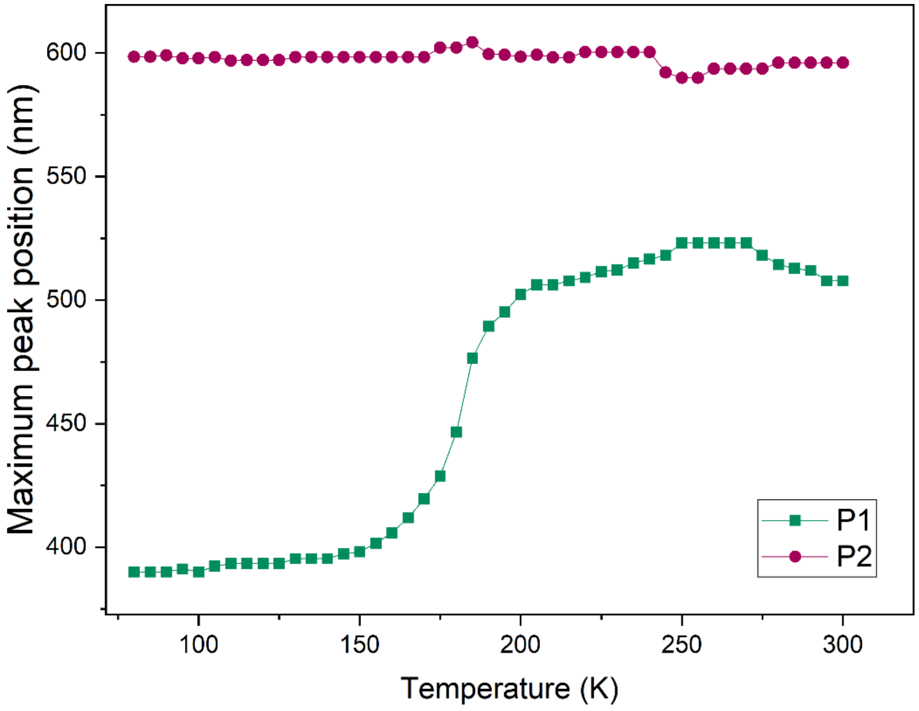

Figure S13. Changes of maximum P_1 and P_2 peak position with temperature.

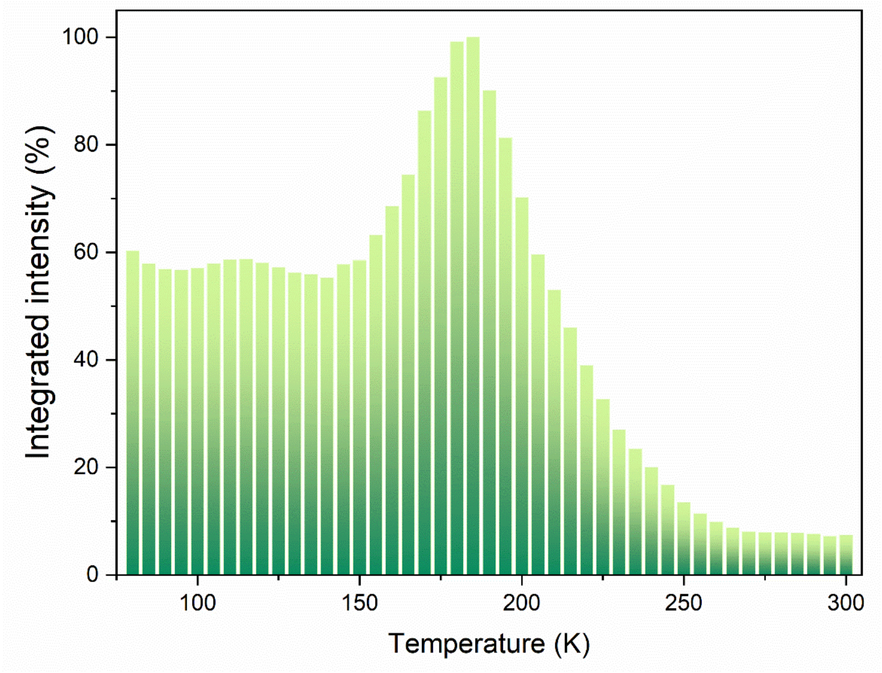

Figure S14. Normalized integrated emission intensity as a function of the temperature of emission band, denoted as P_1, of CPA_2PbCl_4.

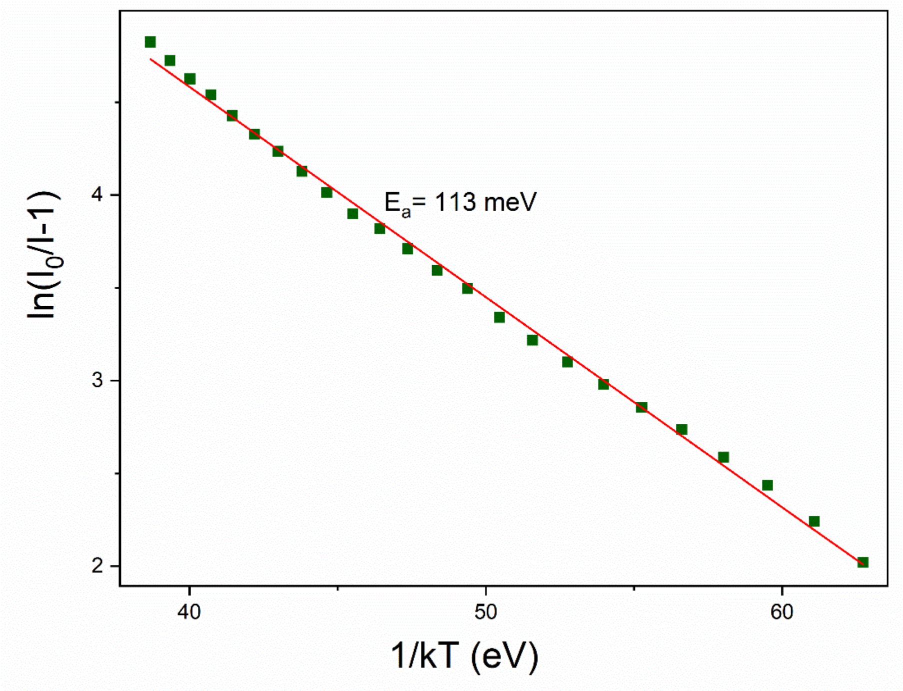

Figure S15. Logarithm of I_0/I-1 as a function of 1/kT, of emission band denoted as P_1 in CPA_2PbCl_4. E_a was extracted by fitting the Arrhenius equation.

SHG

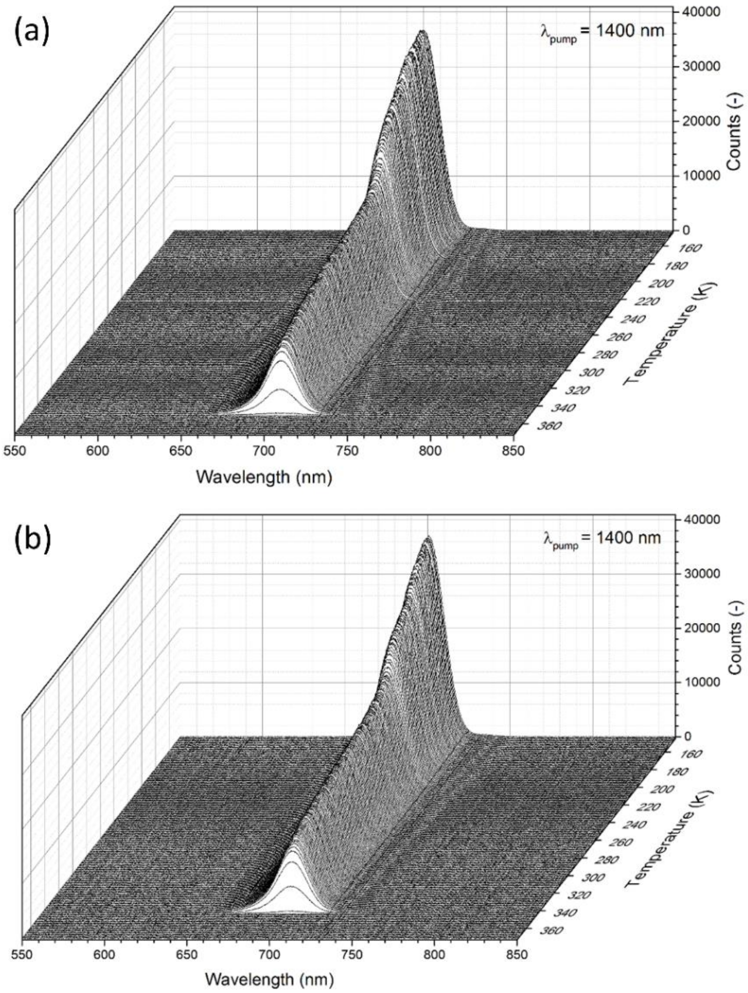

Figure S16. Overlay of experimental SHG spectra obtained upon irradiation with 1400 nm femtosecond laser pulses of CPA_2PbCl_4 for (a) heating in 145–373 K range, (b) cooling in 373–145 K range.

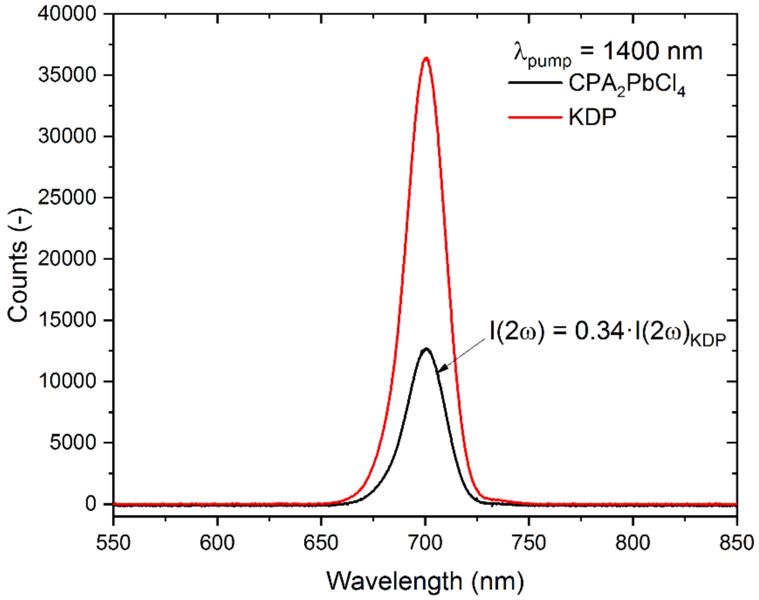

Figure S17. Overlay of SHG trace of CPA_2PbCl_4 with that of KDP obtained upon irradiation with 1400 nm femtosecond laser pulses. SHG traces are normalized to the same integration time.

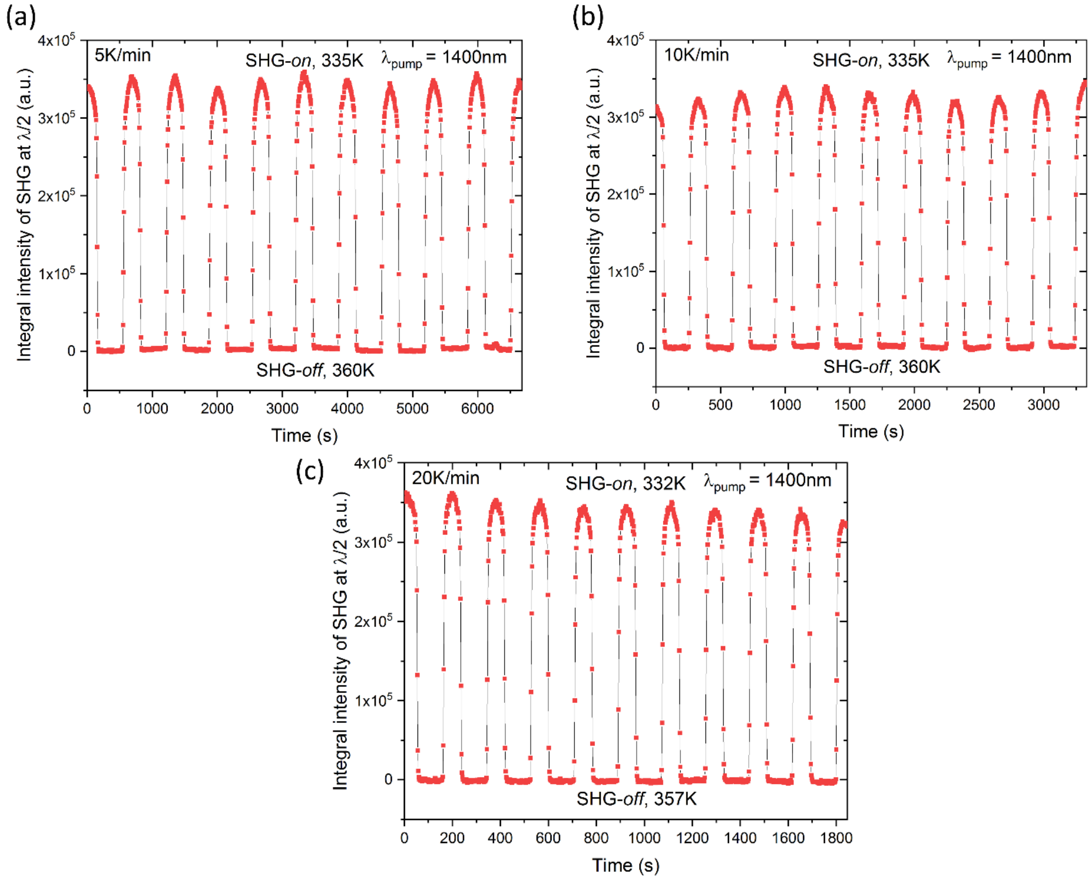

Figure S18. Plots of SHG intensities obtained for temperature switching experiments with (a) d*T*/d*t*=5 $Kmin^{-1}$ between 335 and 360 K, (b) d*T*/d*t*=10 $Kmin^{-1}$ between 335 and 360 K and (a) d*T*/d*t*=20 $Kmin^{-1}$ between 332 and 357 K.

PXRD

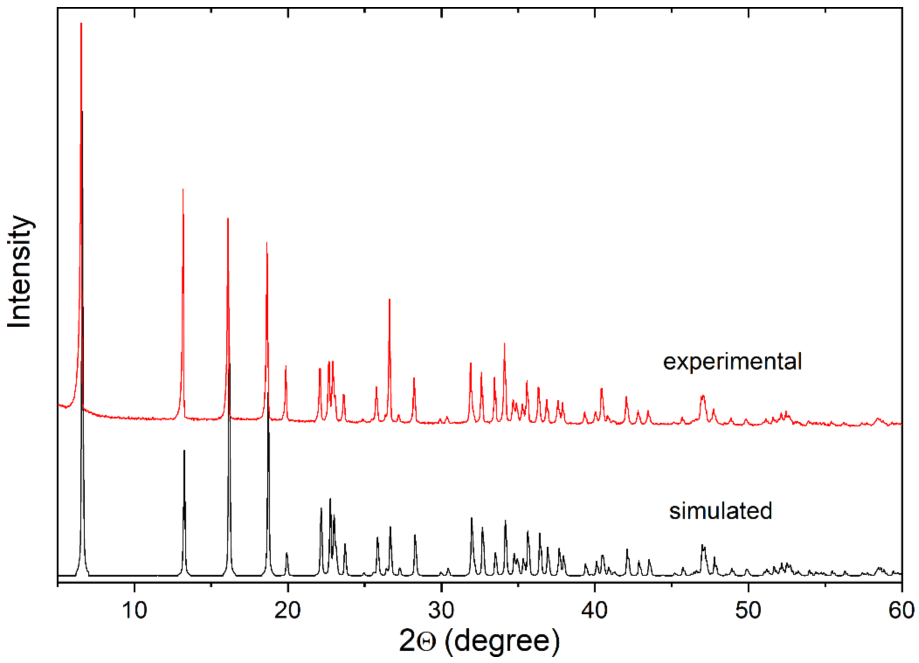

Figure S19. Experimental and simulated RT powder XRD patterns of CPA_2PbCl_4.

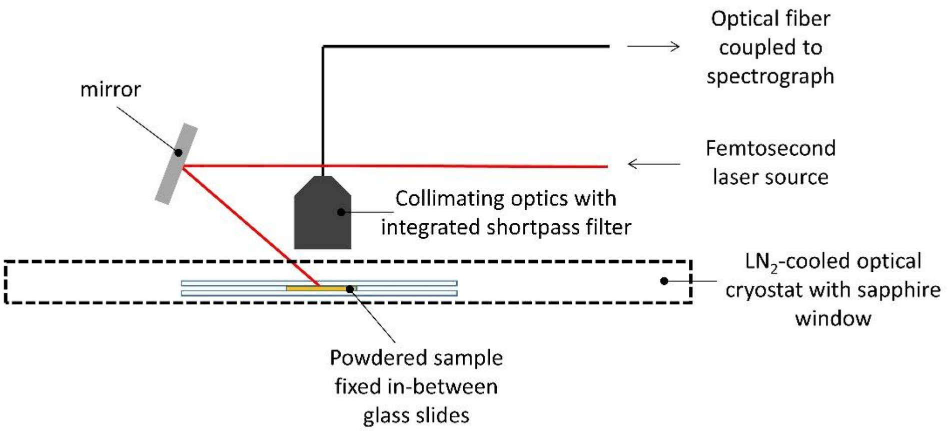

Figure S20. Schematic diagram of the optical setup employed for SHG measurements.
